# Genetics and athletic performance: a systematic SWOT analysis of non-systematic reviews

**DOI:** 10.3389/fgene.2023.1232987

**Published:** 2023-08-09

**Authors:** Magdalena Johanna Konopka, Billy Sperlich, Gerard Rietjens, Maurice Petrus Zeegers

**Affiliations:** ^1^ Care and Public Health Research Institute, Maastricht University, Maastricht, Netherlands; ^2^ Department of Epidemiology, Maastricht University, Maastricht, Netherlands; ^3^ Integrative and Experimental Exercise Science and Training, Institute of Sport Science, University of Würzburg, Würzburg, Germany; ^4^ Human Physiology and Sports Physiotherapy Research Group, Vrije Universiteit Brussel, Brussels, Belgium; ^5^ School of Nutrition and Translational Research in Metabolism, Maastricht University, Maastricht, Netherlands

**Keywords:** athletes, exercise, genetics, genomics, SWOT analysis

## Abstract

Exercise genetics/genomics is a growing research discipline comprising several Strengths and Opportunities but also deals with Weaknesses and Threats. This “systematic SWOT overview of non-systematic reviews” (sSWOT) aimed to identify the Strengths, Weaknesses, Opportunities, and Threats linked to exercise genetics/genomics. A systematic search was conducted in the Medline and Embase databases for non-systematic reviews to provide a comprehensive overview of the current literature/research area. The extracted data was thematically analyzed, coded, and categorized into SWOT clusters. In the 45 included reviews five Strengths, nine Weaknesses, six Opportunities, and three Threats were identified. The cluster of Strengths included “advances in technology”, “empirical evidence”, “growing research discipline”, the “establishment of consortia”, and the “acceptance/accessibility of genetic testing”. The Weaknesses were linked to a “low research quality”, the “complexity of exercise-related traits”, “low generalizability”, “high costs”, “genotype scores”, “reporting bias”, “invasive methods”, “research progress”, and “causality”. The Opportunities comprised of “precision exercise”, “omics”, “multicenter studies”, as well as “genetic testing” as “commercial”-, “screening”-, and “anti-doping” detection tool. The Threats were related to “ethical issues”, “direct-to-consumer genetic testing companies”, and “gene doping”. This overview of the present state of the art research in sport genetics/genomics indicates a field with great potential, while also drawing attention to the necessity for additional advancement in methodological and ethical guidance to mitigate the recognized Weaknesses and Threats. The recognized Strengths and Opportunities substantiate the capability of genetics/genomics to make significant contributions to the performance and wellbeing of athletes.

## 1 Introduction

Current findings approximate the degree of genetic heritability contributing to athletic performance to be around 50%, wherein endurance-related traits demonstrate a range of 44%–68% and strength-related traits present a range of 48%–56%. (Zempo et al., 2017; Miyamoto-Mikami et al., 2018) Accordingly, other factors such as environment, different training methods, diets, etc. account for the remaining variation of performance between athletes. Furthermore, it is important to note that environmental factors have the potential to modulate gene expression, without inducing modifications in the underlying genetic code. This phenomenon, commonly referred to as epigenetics, plays a pivotal role in regulating various physiological processes. (Widmann et al., 2019)

The genetic code is a “set of rules” to define how deoxyribonucleic acid (DNA) is translated into amino acids, the building blocks of proteins. DNA consists of four nucleotides: Adenine, Thymine, Cytosine, and Guanine. (Minchin and Lodge, 2019) A genetic variant describes different variants of a particular DNA sequence, and the most common type involves the substitution of a single nucleotide, known as Single-Nucleotide Variants (SNVs). (Robert and Pelletier, 2018) Genetic variants contribute to different observable characteristics of individuals (i.e., phenotypes or traits) such as muscle fiber distribution.

A good example for describing the involvement of genes in exercise is the ACTN3 gene. This gene codes for the alpha-actinin-3 protein, which is responsible for producing forceful muscle contractions. (Houweling et al., 2018) A stop-codon variant (rs1815739; R577X) results in a non-functional protein (i.e., XX genotype) and individuals carrying this genotype lack the ACTN3 protein. In contrast, the R allele (i.e., RX or RR genotype) results in a functional ACTN3 protein. Interestingly, power and strengths athletes more frequently possess the functional protein when compared to endurance athletes. (Del Coso et al., 2019; Baltazar-Martins et al., 2020) Therefore, the R allele has been linked to power/strengths sports and the XX genotype to endurance sports.

One aim of identifying exercise-related variants is the implementation of “precision exercise”, following precision medicine. (Tanisawa et al., 2020) Precision approaches to training and lifestyle interventions consider the inherent individual differences in genes, environmental factors, and lifestyle, in order to eschew generic and uniform “one size fits all” approaches. Instead, personalized and tailored training regimens and lifestyle interventions aim to optimize performance and/or health outcomes, (Ramaswami et al., 2018), reduce the risk of injury, and identify potential areas of talent in each unique individual. (Montalvo et al., 2017; Ross et al., 2019)

In 1998, the first gene (ACE gene) for explaining athletic performance was identified. (Montgomery et al., 1998) At that time, scientists sought to discover the sports gene. Instead, hundreds of variants were found, which likely altogether contribute to athletic performance. Hence, athletic performance is considered a complex trait regulated by the presence of many variants, gene-by-gene, and gene-by-environment interactions, as well as epigenetic influences ultimately challenging the investigation of complex traits. (Rowe and Tenesa, 2012; Ehlert et al., 2013)

However, the advent of next-generation sequencing techniques and the integration of molecular methodologies have facilitated the emergence of a comprehensive research paradigm, known as “multi-omics.” This approach allows for the examination of complex traits through a holistic lens, thereby enabling a more thorough investigation of biological phenomena. (Sellami et al., 2021) Nowadays, it is well known that intricate molecular networks underlie exercise-related traits and genomics may explain a large amount of the variance observed in exercise-related traits. (Bouchard, 2015; Tanisawa et al., 2020; Ginevičienė et al., 2022)

By 2021, more than 220 variants have been linked to various exercise-related traits (e.g., elite athlete status, endurance, strength, power, speed, recovery, injury, etc.). (Ahmetov and Fedotovskaya, 2015; Ahmetov et al., 2016; Ahmetov et al., 2022; Silva et al., 2022) Moreover, genotype scores and prediction models for personalized training strategies have been developed and implemented. (Guilherme and Lancha, 2020; Pranckeviciene et al., 2021; Yang et al., 2021) In recent times, private direct-to-consumer genetic testing companies sell online sport-related genetic tests. (Vlahovich et al., 2017) These companies employ marketing claims, such as the following: “To reach the top in the sporting world, it is not enough to train hard; you have to train intelligently, to know yourself and how genetics influences sport is the best starting point” (www.24genetics.com). These assertions may seem appealing to athletes seeking to improve their performance. However, the field of exercise genetics/genomics is not free of challenges and potential drawbacks. These include issues such as small sample sizes, inconsistent findings, and ethical concerns surrounding gene doping involving the manipulation of genetic material to enhance athletic performance. (Cantelmo et al., 2020) In conclusion, exercise genetics deals with many “Strengths”, “Weaknesses”, “Opportunities”, and “Threats” (SWOTs).

A SWOT analysis identifies current strengths, future opportunities, areas of weakness that require attention, and potential threats. It allows to assess the progress of a certain topic at a certain point in time, identifies research gaps, and offers future directions. (Helms and Nixon, 2010) As such, a SWOT analysis is an efficient and powerful tool to generate meaningful information for strategic decision-making and to guide the development of future effective action plans. (de-Madaria et al., 2022) In addition, numerous systematic (e.g., systematic reviews/meta-analysis) and non-systematic (e.g., opinion papers, commentaries, narrative reviews, scoping reviews) publications have summarized the evidence of exercise genetics/genomics. Non-systematic reviews are considered an important tool for examining different theoretical conceptualizations, constructs, and/or relationships. (Baumeister and Prinstein, 2013) The fusion of existing reviews allows a synthesis of current evidence thereby stimulating a broader comprehension of research questions. (Gates et al., 2020) To our knowledge, within the exercise genetic/genomic literature, no “overview of non-systematic reviews” about “Strengths”, “Weaknesses”, “Opportunities”, and “Threats” exists. We therefore aimed to comprise an overview of non-systematic reviews to highlight the current Strengths, Weaknesses, Opportunities, and Threats of exercise genetics/genomics.

## 2 Materials and methods

### 2.1 Study design, reporting guidelines, research protocol

In this “systematic SWOT overview of non-systematic reviews” (sSWOT), we summarized the i) Strengths, ii) Weaknesses, iii) Opportunities, and iv) Threats of this topic. The “Reporting guideline for overviews of reviews of healthcare interventions: development of the PRIOR statement guidelines” has recently been published (2022). (Gates et al., 2022) Since this guideline was established for overviews of systematic reviews focusing on healthcare interventions these standards are not applicable for our purpose. Hence, we followed the “reporting checklist for overviews of reviews” developed by Onishi and Furukawa in 2016 (Onishi and Furukawa, 2016) (see electronic Supplementary Material (ESM) 1 for the checklist). Sometimes, overview of reviews are also referred to as “meta-reviews”, “reviews of reviews,” or “umbrella reviews”. (Tsagris and Fragkos, 2016) The research protocol is based on the “preferred reporting items for systematic review and meta-analysis protocols” (PRISMA-P) and accessible at the open science framework (DOI 10.17605/OSF.IO/TVKUM).

### 2.2 Search strategy and eligibility criteria

We performed a systematic literature search, in accordance with the PRISMA-S guidelines (Rethlefsen et al., 2021) on 19^th^ of May 2022 by searching the Medline and Embase databases. The search was updated on 8^th^ of January 2023. We employed the following search terms: athlete, athletic, exercise, genetics, and genomics. We applied the “review” filter to increase the precision of the search results (Franco et al., 2020; Salvador-Oliván et al., 2021) and limited the search to “title” and “abstracts” only with no date restriction. ESM 2 displays the full search strategy. Non-systematic reviews (e.g., narrative and scoping reviews, commentaries, or opinion papers) in English with the topic of exercise genetics/genomics were eligible for inclusion. We excluded reviews aiming at specific sport disciplines and/or genetic variants or genes (e.g., injury, nutrition, speed, ACE, ACTN3, etc.). Furthermore, systematic reviews, primary studies, animal studies, conference abstracts, and case studies were also excluded. The retrieved reviews were extracted to Endnote (Clarivate Analytics, Philadelphia, Pennsylvania, United States) and automatically screened for duplicates. The title and abstract were screened by one reviewer (MK). In case that a review seemed appropriate for inclusion, the full text was read and when the full text was not available, we contacted the authors. Finally, all included reviews were screened for cross-references to minimize the chance of missing relevant reviews.

### 2.3 Data extraction and data items

The main investigator (MK) extracted the data using Excel, with an 8-week wash out period. The two data extraction sheets were compared and in case of detection of major differences in extracted themes, the reviews of interest were read once more. We extracted the following items: first author’s name, publication year, title, Strengths, Weaknesses, Opportunities, and Threats. Risk of bias and certainty assessment of the non-systematic reviews was not applicable.

### 2.4 Data synthesis

The extracted data was thematically analyzed and structured into SWOTs. (Braun and Clarke, 2006) The coding strategy consisted of three stages: i) initial coding - remaining open to all possible themes indicated by initial readings of the reviews, (Charmaz, 2006; Corbin and Strauss, 2008), ii) focused coding—categorizing the data inductively based on thematic similarity, (Charmaz, 2006), and finally iii) theoretical coding - integrating thematic categories. (Saldaña, 2021) In the first cycle, open descriptive codes derived directly from the articles (e.g., integration of novel algorithm approaches, results from candidate gene study designs, statistical shortcomings such as multiple testing). Full sentences were treated as unique items on the data extraction sheet and coded to generate a range of information. In a second step, a focused thematic analysis identified patterns among the literature to confirm links between the openly coded data. Thematic phrases (e.g., advances in technology, study design, methodology) were consequently developed and reapplied to coded items in the data extraction sheet. In the third step, the thematic phrases were ordered according to frequency and aggregated into one of the four categories reflecting the scope of this overview: i) Strengths, ii) Weaknesses, iii) Opportunities, and iv) Threats.

## 3 Results

### 3.1 Review selection

**FIGURE 1:**
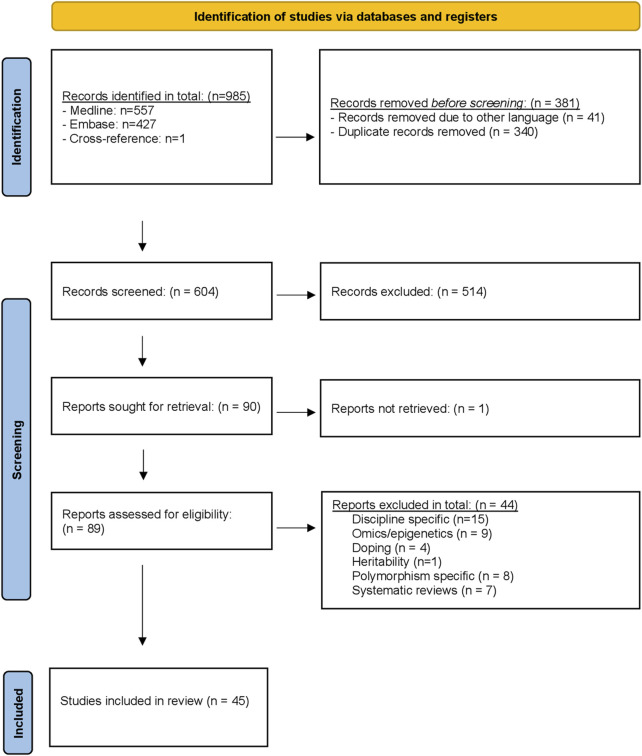
PRISMA flow diagram of study selection.

The search retrieved 984 records and we identified one additional cross-reference. (Ahmetov et al., 2022) After eliminating 381 records (340 duplicates and 41 non-English) 604 records remained. Based on title and abstract reading, we found 90 reviews eligible for inclusion of which one record could not be retrieved even after contacting the author. (Patel and Greydanus, 2002) Finally, 45 records were included in this overview based on full text reading. (Tanisawa et al., 2020; Sellami et al., 2021; Bouchard, 2015; Ahmetov and Fedotovskaya, 2015; Vlahovich et al., 2017; Bray, 2000; Brutsaert and Parra, 2006; Sharp, 2008; McNamee et al., 2009; Ostrander et al., 2009; Wackerhage et al., 2009; Lippi et al., 2010; Rankinen et al., 2010; Bouchard, 2011; Bouchard et al., 2011; Eynon et al., 2011; Hagberg et al., 2011; Roth et al., 2012; Guth and Roth, 2013; Pérusse et al., 2013; Wang et al., 2013; Breitbach et al., 2014; Wolfarth et al., 2014; Bouchard et al., 2015; Loos et al., 2015; Webborn et al., 2015; Gibson, 2016; Mattsson et al., 2016; Pitsiladis et al., 2016; Sarzynski et al., 2016; Wang et al., 2016; Yan et al., 2016; Moran and Pitsiladis, 2017; Vellers et al., 2018; Landen et al., 2019; Pickering and Kiely, 2019; Pickering et al., 2019; Gomes et al., 2020; Gray and Semsarian, 2020; Naureen et al., 2020; Griswold et al., 2021; Wang and Ashokan, 2021; Kim et al., 2022) The flow chart of the study selection is presented in Figure 1. The main reasons for exclusion were “discipline specific” reviews (n = 15), (Eynon et al., 2013; Maffulli et al., 2013; Tucker et al., 2013; Vancini et al., 2014; Heffernan et al., 2015; Lundby et al., 2017; Costello et al., 2018; Southward et al., 2018; Brazier et al., 2019; Herbert et al., 2019; Antrobus et al., 2021; Barreto et al., 2021; Meyler et al., 2021; Cabrera-Serrano and Ravenscroft, 2022; Mareddy et al., 2022), “omics/epigenetics” (n = 9), (Ehlert et al., 2013; Bassini and Cameron, 2014; Kennedy et al., 2018; Hill et al., 2019; Polli et al., 2019; Hall et al., 2020; Kelly et al., 2020; Malsagova et al., 2021; Nieman, 2021), “doping” (n = 4), (Unal and Ozer Unal, 2004; Gaffney and Parisotto, 2007; Schneider et al., 2012; Fischetto and Bermon, 2013), “heritability” related reviews (n = 1), (Zhang and Speakman, 2019), “polymorphism specific” reviews (n = 8), (Gordon et al., 2005; Sarpeshkar and Bentley, 2010; Angelopoulos et al., 2011; Collins et al., 2016; Venezia and Roth, 2016; Pickering and Kiely, 2017; Del Coso et al., 2019; van de Vegte et al., 2019), and “systematic reviews” (n = 7). (Rankinen et al., 2006; Alvarez-Romero et al., 2021; Balberova et al., 2021; Ahmetov et al., 2022; Ginevičienė et al., 2022; Konopka et al., 2022; Mohan, 2022) ESM 3 contains the reasoning for exclusion. ESM 4 presents the key characteristics of the reviews including the thematic phrases (i.e., themes) identified in the second analysis cycle. The full content analysis (cycle 1–3) is displayed in ESM 5–8. Overall, within the four clusters, five themes related to Strengths, nine Weaknesses, six Opportunities, and three Threats were identified (Table 1).

**TABLE 1:** Summary of themes, clustered into SWOTs.

Strengths (n = 5)	Weaknesses (n=9)
• Advances in technology (n = 31)	• Quality of studies, internal validity (n = 31)
• Empirical evidence (n = 25)	• Complexity of exercise-related traits (n = 28)
• Growing research field (n = 8)	• Generalizability, external validity (n = 13)
• Consortia/consensus statements/guidelines (n = 8)	• Costs (n = 8)
• Acceptance/accessibility of genetic testing (n = 2)	• Genotype scores (n = 8)
	• Reporting bias (n = 4)
	• Invasive methods (n = 4)
	• Research progress (n = 2)
	• Causality (n = 1)

Opportunities (n = 6)	Threats (n = 3)
• Precision exercise/gene profiling (n = 25)	• Ethics of genetic testing (n = 13)
• Omics/technology (n = 25)	• Direct-to-consumer genetic testing companies (n = 11)
• Multicentre studies (n = 18)	• Doping/gene editing (n = 6)
• Screening/therapy (n = 12)	
• Anti-doping detection (n = 2)	
• Commercial use (n = 1)	

n = frequency of themes identified.

### 3.2 Cluster: strengths

#### 3.2.1 Theme: advances in technology

“Advances in technology” was identified by the thematic analysis and clustered as a Strength. According to several reviewers, the completion of the Human Genome Project in 2003 and HapMap enabled to analyze complex traits. (Bouchard et al., 2015; Yan et al., 2016; Pickering and Kiely, 2019; Gomes et al., 2020; Griswold et al., 2021) Advances in methodology and technology, such as the development of genome-wide association studies (i.e., hypothesis free approaches), the use of high-throughput sequencing technologies (fast and inexpensive), as well as innovative analytical approaches (e.g., artificial intelligence, machine learning) contributed to the progress of exercise genomics/genetics. (Bray, 2000; Brutsaert and Parra, 2006; Sharp, 2008; McNamee et al., 2009; Lippi et al., 2010; Bouchard, 2011; Bouchard et al., 2011; Eynon et al., 2011; Pérusse et al., 2013; Wang et al., 2013; Breitbach et al., 2014; Wolfarth et al., 2014; Ahmetov and Fedotovskaya, 2015; Bouchard, 2015; Bouchard et al., 2015; Loos et al., 2015; Webborn et al., 2015; Gibson, 2016; Mattsson et al., 2016; Sarzynski et al., 2016; Wang et al., 2016; Yan et al., 2016; Moran and Pitsiladis, 2017; Vlahovich et al., 2017; Vellers et al., 2018; Landen et al., 2019; Pickering et al., 2019; Pickering and Kiely, 2019; Gomes et al., 2020; Tanisawa et al., 2020; Griswold et al., 2021)

#### 3.2.2 Theme: empirical evidence

The thematic analysis also identified “empirical evidence” as one of the Strengths of exercise genetics/genomics. Highlighted by many authors, the heritability estimates of exercise-related traits vary between 40% and 70%, depending on the trait under investigation. (Brutsaert and Parra, 2006; Ostrander et al., 2009; Wackerhage et al., 2009; Lippi et al., 2010; Bouchard, 2011; Eynon et al., 2011; Guth and Roth, 2013; Wang et al., 2013; Breitbach et al., 2014; Ahmetov and Fedotovskaya, 2015; Bouchard et al., 2015; Wang et al., 2016; Yan et al., 2016; Moran and Pitsiladis, 2017; Vellers et al., 2018; Landen et al., 2019; Pickering et al., 2019; Gomes et al., 2020; Naureen et al., 2020; Tanisawa et al., 2020; Griswold et al., 2021; Wang and Ashokan, 2021; Kim et al., 2022; Varillas-Delgado et al., 2022; Viecelli and Ewald, 2022) For instance, the heritability for “achieving elite athletic status” has been estimated approximately 66%, (Guth and Roth, 2013; Ahmetov and Fedotovskaya, 2015; Varillas-Delgado et al., 2022), the “change in maximal oxygen uptake in response to training” around 50%, (Brutsaert and Parra, 2006; Wackerhage et al., 2009; Bouchard, 2011; Bouchard et al., 2015; Moran and Pitsiladis, 2017; Gomes et al., 2020; Griswold et al., 2021; Wang and Ashokan, 2021; Kim et al., 2022), and the “transmissibility of muscle mass to be greater than 90%.” (Viecelli and Ewald, 2022) According to several authors, “more than 200 performance enhancing polymorphisms exist.” (Ostrander et al., 2009; Eynon et al., 2011; Guth and Roth, 2013; Wang et al., 2013; Wang et al., 2016; Yan et al., 2016; Naureen et al., 2020) Thus, empirical evidence (e.g., high heritability estimates) supports the role of genetics within athletic performance.

#### 3.2.3 Theme: growing research field

Several reviewers described exercise genetics/genomics as a “growing research field” which therefore has been clustered into Strengths. (Brutsaert and Parra, 2006; Wolfarth et al., 2014; Ahmetov and Fedotovskaya, 2015; Loos et al., 2015; Gibson, 2016; Vlahovich et al., 2017; Pickering and Kiely, 2019; Varillas-Delgado et al., 2022. For example, Ahmetov and Fedotovskaya (2015) reported: “By the end of 2014, the total number of DNA polymorphisms related to exercise genetics was 120. Most of these polymorphisms (70%) were discovered in the last 5 years (2010–2014), indicating a growing interest in the field of sports genomics.” (Ahmetov and Fedotovskaya, 2015)

#### 3.2.4 Theme: consortia/consensus statements/guidelines

“Consortia, consensus statements and guidelines” were identified by the present sSWOT as Strengths. Several narratives mentioned the establishment of “international, large-scale, and well-funded” consortia (e.g., www.athlomeconsortium.org). (Wang et al., 2013; Pitsiladis et al., 2016; Wang et al., 2016; Vlahovich et al., 2017; Gomes et al., 2020; Tanisawa et al., 2020; Griswold et al., 2021; Sellami et al., 2021) According to these findings, the “interdisciplinary consortia” but also the “consensus statements” and “guidelines” for conducting good exercise genetic/genomic research may contribute to an increased research quality providing sufficient scientific evidence. (Tanisawa et al., 2020)

#### 3.2.5 Theme: acceptance/accessibility of genetic performance testing

“Acceptance and accessibility of genetic performance testing” was additionally classified as Strengths. The thematic analysis identified one report emphasizing “athletes are generally open for genetic performance testing” (Pickering and Kiely, 2019) and, in addition, genetic testing was described as “non-invasive and quick method for performance testing” which may contribute to generally accepting genetic testing within a sporting context. (Lippi et al., 2010)

### 3.3 Cluster: weaknesses

#### 3.3.1 Theme: quality of studies

Most Weaknesses identified by the thematic analysis were related to methodological shortcomings resulting from “low quality studies.” (Brutsaert and Parra, 2006; Ostrander et al., 2009; Wackerhage et al., 2009; Rankinen et al., 2010; Bouchard, 2011; Bouchard et al., 2011; Eynon et al., 2011; Hagberg et al., 2011; Roth et al., 2012; Guth and Roth, 2013; Wang et al., 2013; Breitbach et al., 2014; Ahmetov and Fedotovskaya, 2015; Bouchard, 2015; Loos et al., 2015; Webborn et al., 2015; Gibson, 2016; Mattsson et al., 2016; Pitsiladis et al., 2016; Wang et al., 2016; Moran and Pitsiladis, 2017; Vlahovich et al., 2017; Vellers et al., 2018; Landen et al., 2019; Pickering et al., 2019; Gomes et al., 2020; Naureen et al., 2020; Tanisawa et al., 2020; Griswold et al., 2021; Kim et al., 2022; Varillas-Delgado et al., 2022) Numerous scholars have contended that the present body of knowledge in the field of sport genetics/genomics is primarily rooted in investigations of candidate genes (i.e., research designed to test a priori hypotheses using case-control designs), which typically involve limited sample sizes and, as a result, frequently exhibit insufficient statistical power. (Brutsaert and Parra, 2006; Wang et al., 2013; Ahmetov and Fedotovskaya, 2015; Loos et al., 2015; Mattsson et al., 2016; Pitsiladis et al., 2016; Yan et al., 2016; Moran and Pitsiladis, 2017; Vlahovich et al., 2017) Some authors acknowledged that candidate gene studies produced “inconclusive results” or “false positives” (Wang et al., 2013; Ahmetov and Fedotovskaya, 2015; Loos et al., 2015; Gibson, 2016; Mattsson et al., 2016; Moran and Pitsiladis, 2017; Varillas-Delgado et al., 2022). Furthermore, Kim et al. (2020) (Kim et al., 2022) highlighted that “only a handful of genome-wide association studies” have been performed in a exercise science context, and according to Griswold et al., (2021) (Griswold et al., 2021) “even the largest genome-wide association study to date in elite endurance athletes (a total of 1,520 athletes and 2,760 controls) was not able to identify any significantly associated genetic markers”. Other reviewers argued that “inconsistent study protocols”, (Vellers et al., 2018; Gomes et al., 2020; Kim et al., 2022), poor “definitions and measurements of phenotypes”, (Bouchard, 2011; Eynon et al., 2011; Roth et al., 2012; Ahmetov and Fedotovskaya, 2015; Gibson, 2016; Mattsson et al., 2016; Wang et al., 2016; Vellers et al., 2018; Gomes et al., 2020; Naureen et al., 2020; Tanisawa et al., 2020; Griswold et al., 2021; Varillas-Delgado et al., 2022), and poor “classifications of sport disciplines and performance level” (Ostrander et al., 2009; Eynon et al., 2011; Breitbach et al., 2014; Naureen et al., 2020) would increase the “(phenotypic) heterogeneity.” (Ostrander et al., 2009; Bouchard et al., 2011; Eynon et al., 2011; Breitbach et al., 2014; Gibson, 2016; Wang et al., 2016; Moran and Pitsiladis, 2017; Vlahovich et al., 2017; Naureen et al., 2020; Griswold et al., 2021; Varillas-Delgado et al., 2022) Finally, some authors mentioned that the employment of inappropriate (sedentary) control groups, (Bouchard et al., 2011; Moran and Pitsiladis, 2017; Varillas-Delgado et al., 2022), the lack of blinding, (Gibson, 2016), not accounting for multiple testing, (Rankinen et al., 2010; Bouchard, 2011; Hagberg et al., 2011; Roth et al., 2012; Wang et al., 2013; Wang et al., 2016), as well as a low genotyping quality and errors (Rankinen et al., 2010; Gibson, 2016) may contribute to a “low internal validity” of exercise genetic/genomic studies.

#### 3.3.2 Themes: complexity of exercise-related traits, generalizability

Many reviewers described the “complexity of exercise-related traits”, which was identified as a Weakness in the present overview. (Bray, 2000; Brutsaert and Parra, 2006; Wackerhage et al., 2009; Lippi et al., 2010; Bouchard, 2011; Bouchard et al., 2011; Eynon et al., 2011; Roth et al., 2012; Guth and Roth, 2013; Wang et al., 2013; Wolfarth et al., 2014; Ahmetov and Fedotovskaya, 2015; Bouchard, 2015; Loos et al., 2015; Gibson, 2016; Mattsson et al., 2016; Wang et al., 2016; Yan et al., 2016; Moran and Pitsiladis, 2017; Vellers et al., 2018; Landen et al., 2019; Pickering et al., 2019; Pickering and Kiely, 2019; Gomes et al., 2020; Tanisawa et al., 2020; Griswold et al., 2021; Varillas-Delgado et al., 2022) Multifaceted characteristics, such as exercise-related traits, are considered polygenic in nature, meaning that they are influenced by numerous variants, gene-gene and gene-environment interactions, epigenetic modifications, and other factors. The individual impact of each variant is typically minor. A consensus among several authors is that current research on exercise genetics/genomics has primarily concentrated on exploring common genetic variants, while paying less attention to rare genetic variants. (Bouchard, 2011; Roth et al., 2012; Wang et al., 2013; Mattsson et al., 2016; Moran and Pitsiladis, 2017; Pickering et al., 2019; Gomes et al., 2020; Griswold et al., 2021) For example, Bouchard et al., (Bouchard, 2011), among others, stated that “rare variants are most likely the high-impact variants” but challenging to uncover. In addition, other types of genomic variation, such as structural variations (e.g., copy number variations, insertions and deletions) and non-coding RNA (e.g., micro-RNA) also contribute to exercise-related traits but “would not be captured by current genotyping methods”. (Bouchard, 2011; Bouchard et al., 2011; Roth et al., 2012; Wang et al., 2013; Vellers et al., 2018) The “generalizability” of exercise genetic/genomic study results has also been identified as Weakness by the thematic analysis. (Rankinen et al., 2010; Bouchard et al., 2011; Eynon et al., 2011; Ahmetov and Fedotovskaya, 2015; Webborn et al., 2015; Mattsson et al., 2016; Wang et al., 2016; Vlahovich et al., 2017; Landen et al., 2019; Pickering et al., 2019; Naureen et al., 2020; Griswold et al., 2021; Kim et al., 2022) Several reviewers mentioned a low generalizability due to “sex imbalances”, (Mattsson et al., 2016; Vlahovich et al., 2017; Landen et al., 2019; Kim et al., 2022), or “lack of population stratification.” (Rankinen et al., 2010; Bouchard et al., 2011; Eynon et al., 2011; Ahmetov and Fedotovskaya, 2015; Mattsson et al., 2016; Wang et al., 2016; Landen et al., 2019; Naureen et al., 2020; Griswold et al., 2021; Kim et al., 2022)

#### 3.3.3 Themes: costs, genotype scores, reporting bias

The sSWOT analysis additionally identified a Weakness related to the significant “costs” of genetic research on one hand, and the inadequate funding available on the other hand. (Brutsaert and Parra, 2006; Wackerhage et al., 2009; Lippi et al., 2010; Hagberg et al., 2011; Roth et al., 2012; Loos et al., 2015; Gibson, 2016; Sarzynski et al., 2016) Another Weakness was linked to “genotype scores”. (Eynon et al., 2011; Wang et al., 2013; Breitbach et al., 2014; Webborn et al., 2015; Mattsson et al., 2016; Pickering et al., 2019; Naureen et al., 2020; Varillas-Delgado et al., 2022) According to some reviewers, genotype scores in an exercise science context have “zero predictive capability”, “limited real world sensitivity and specificity, (Wang et al., 2016), and “lack appropriate weighing factors”. (Varillas-Delgado et al., 2022) The content analysis additionally revealed “reporting bias” as a Weakness. (Rankinen et al., 2010; Eynon et al., 2011; Wang et al., 2013; Gibson, 2016) For example, Rankinen et al. (2019) stated: “very few negative studies reach publication.” (Rankinen et al., 2010)

#### 3.3.4 Themes: invasive methods, research progress, causality

The thematic analysis identified three more Weaknesses related to “invasive methods”, (Eynon et al., 2011; Gibson, 2016; Mattsson et al., 2016; Pickering and Kiely, 2019), “research progress”, (Wang et al., 2013; Yan et al., 2016), and “causality”. (Mattsson et al., 2016) Some authors highlighted that more “invasive methods” (e.g., biopsies) may be necessary to entangle the complexity of exercise genetics/genomics. (Eynon et al., 2011; Gibson, 2016; Mattsson et al., 2016; Pickering and Kiely, 2019) Further, based on the report of Wang et al. (2013) there has been “limited research progress” and output of the consortia and multi-center studies. (Wang et al., 2013; Yan et al., 2016) Finally, Mattson et al. (2016) stated: “It is important to emphasize that genetic association, regardless of how robust, does not infer causality.” (Mattsson et al., 2016)

### 3.4 Cluster: opportunities

#### 3.4.1 Theme: precision exercise/gene profiling

The present sSWOT identified “precision exercise” approaches based on “genetic profiling” as an Opportunity as highlighted by many reviewers. (Sharp, 2008; Lippi et al., 2010; Bouchard, 2011; Eynon et al., 2011; Guth and Roth, 2013; Pérusse et al., 2013; Wolfarth et al., 2014; Ahmetov and Fedotovskaya, 2015; Bouchard et al., 2015; Loos et al., 2015; Mattsson et al., 2016; Pitsiladis et al., 2016; Sarzynski et al., 2016; Vlahovich et al., 2017; Landen et al., 2019; Pickering et al., 2019; Pickering and Kiely, 2019; Gomes et al., 2020; Naureen et al., 2020; Tanisawa et al., 2020; Griswold et al., 2021; Sellami et al., 2021; Wang and Ashokan, 2021; Kim et al., 2022; Varillas-Delgado et al., 2022). According to these authors, exercise genetics/genomics has the potential to “optimize and maximize physical performance” (Sellami et al., 2021) by implementing precision exercise methods. Several reviewers suggested, “once genetic associations have been identified in a robust and valid manner”, (Mattsson et al., 2016), improved genotype scores may be developed by integrating (among others) “data science”, “algorithms”, or “machine learning”. (Eynon et al., 2011; Pickering et al., 2019; Pickering and Kiely, 2019; Kim et al., 2022) By using “genetic profiling”, coaches and athletes might be able “to match individuals to the type of training to which they are most suited, and from which they will elicit the greatest adaptations”, according to Pickering and others (2019). (Pickering et al., 2019). Some reviewers also mentioned a possible impact on “injury risks”, (Guth and Roth, 2013; Varillas-Delgado et al., 2022), “recovery periods”, (Sharp, 2008; Guth and Roth, 2013; Ahmetov and Fedotovskaya, 2015; Pitsiladis et al., 2016; Naureen et al., 2020; Varillas-Delgado et al., 2022), and “talent identification” (Pickering et al., 2019) as a field of application.

#### 3.4.2 Theme: omics/technology

“Omics and technology” were identified as Opportunities in the current overview. (Bray, 2000; Brutsaert and Parra, 2006; Bouchard, 2011; Bouchard et al., 2011; Eynon et al., 2011; Pérusse et al., 2013; Wang et al., 2013; Wolfarth et al., 2014; Ahmetov and Fedotovskaya, 2015; Bouchard, 2015; Bouchard et al., 2015; Gibson, 2016; Mattsson et al., 2016; Pitsiladis et al., 2016; Sarzynski et al., 2016; Wang et al., 2016; Moran and Pitsiladis, 2017; Landen et al., 2019; Pickering and Kiely, 2019; Gomes et al., 2020; Tanisawa et al., 2020; Griswold et al., 2021; Sellami et al., 2021; Kim et al., 2022; Varillas-Delgado et al., 2022) Many reviewers agreed that by integrating “multi-omics” research approaches (e.g., epigenomics, transcriptomics, proteomics, metabolomics, etc.) more complex molecular pathways could be analyzed, which may allow to “explore a more comprehensive picture of athletic performance and its exercise-related traits”. (Bouchard, 2011; Bouchard et al., 2011; Eynon et al., 2011; Pérusse et al., 2013; Wolfarth et al., 2014; Ahmetov and Fedotovskaya, 2015; Bouchard, 2015; Bouchard et al., 2015; Gibson, 2016; Mattsson et al., 2016; Sarzynski et al., 2016; Wang et al., 2016; Moran and Pitsiladis, 2017; Landen et al., 2019; Pickering and Kiely, 2019; Gomes et al., 2020; Tanisawa et al., 2020; Sellami et al., 2021; Kim et al., 2022; Varillas-Delgado et al., 2022) In addition, others’ argued that “whole genome/exome sequencing” and “RNA expression profiling” next to other cutting-edge technologies would provide promising approaches in future to explore rare variants, copy number variation, and other structural variants. (Bray, 2000; Brutsaert and Parra, 2006; Bouchard, 2011; Wang et al., 2013; Ahmetov and Fedotovskaya, 2015; Bouchard, 2015; Gibson, 2016; Pitsiladis et al., 2016; Vellers et al., 2018; Pickering and Kiely, 2019; Tanisawa et al., 2020; Sellami et al., 2021; Varillas-Delgado et al., 2022) For example, according to Griswold et al. (2021) both advances in technologies together with multi-omics approaches will be necessary to “truly have a holistic view of genomics.” (Griswold et al., 2021)

#### 3.4.3 Theme: multicenter studies

Several narratives described “multicenter (genome-wide association) studies” as an Opportunity for detecting new variants by increasing the studies’ statistical power. (Bouchard, 2011; Eynon et al., 2011; Hagberg et al., 2011; Wang et al., 2013; Ahmetov and Fedotovskaya, 2015; Bouchard, 2015; Loos et al., 2015; Mattsson et al., 2016; Wang et al., 2016; Moran and Pitsiladis, 2017; Vlahovich et al., 2017; Vellers et al., 2018; Landen et al., 2019; Gomes et al., 2020; Griswold et al., 2021; Sellami et al., 2021; Kim et al., 2022; Varillas-Delgado et al., 2022) For example, the large scale and multicenter Athlome Project was launched in 2015 with the goal to “generate an ethically sound environment, interest, and capacity needed to develop the specialist knowledge to inform personalized training and injury prevention, as well as doping detection”. (Pitsiladis et al., 2016) Wang and others (2016) stated: “This unique collaborative initiative has the greatest chance to succeed where individual efforts have failed, to increase our understanding of the biology of exercise performance and related performance traits.” (Wang et al., 2016)

#### 3.4.4 Themes: screening/therapy, anti-doping, commercial use

Other themes identified by the thematic analysis and highlighted by various reviewers were linked to genetic “screening, prevention, and therapy”—which may contribute to the “health of athletes”. (Sharp, 2008; McNamee et al., 2009; Wackerhage et al., 2009; Lippi et al., 2010; Bouchard, 2011; Pérusse et al., 2013; Breitbach et al., 2014; Bouchard, 2015; Vlahovich et al., 2017; Pickering and Kiely, 2020; Tanisawa et al., 2020; Griswold et al., 2021) For example, according to the “Sport and Exercise Genomics” consensus statement from 2020, (Tanisawa et al., 2020), genetic testing “may have a potential role in cardiac screening”. Gene editing may also offer therapeutic opportunities, for instance gene editing may help to heal ligament and tendon injuries. (Lippi et al., 2010; Tanisawa et al., 2020) Additionally, Lippi et al. (2010) stated: “genetic testing might be helpful in the anti-doping context”, which was also raised by Varillas-Delgado and others (2022). (Lippi et al., 2010; Varillas-Delgado et al., 2022). Finally, according to Tanisawa et al. (2020) exercise genetics/genomics has the potential for “commercial use”, which was also an identified Opportunity by the current sSWOT. (Tanisawa et al., 2020)

### 3.5 Cluster: threats

#### 3.5.1 Theme: ethics of genetics testing

One Threat identified by the thematic analysis was related to “ethical concerns” of genetic testing as raised by many reviewers. (McNamee et al., 2009; Wackerhage et al., 2009; Guth and Roth, 2013; Breitbach et al., 2014; Webborn et al., 2015; Pitsiladis et al., 2016; Sarzynski et al., 2016; Wang et al., 2016; Vlahovich et al., 2017; Pickering et al., 2019; Gray and Semsarian, 2020; Naureen et al., 2020; Tanisawa et al., 2020) For instance, athletes must give informed consent when participating in genetic testing, which may already be problematic in adults—but is even more challenging in youth. (Guth and Roth, 2013; Wang et al., 2016; Vlahovich et al., 2017) According to the consensus statement concerning genetic testing from 2015, (Webborn et al., 2015), athletes must have received “sufficient relevant information to understand the risks, benefits, limitations, and implications of the genetic test”. In addition, several authors raised concerns about “autonomy”, (McNamee et al., 2009; Breitbach et al., 2014), “privacy”, (Webborn et al., 2015; Vlahovich et al., 2017), “reidentification”, (Pitsiladis et al., 2016; Tanisawa et al., 2020), “confidentiality”, (McNamee et al., 2009; Vlahovich et al., 2017), “social stigma” and “discrimination” (Vlahovich et al., 2017)—issues associated with genetic testing of athletes. (Webborn et al., 2015; Pitsiladis et al., 2016; Vlahovich et al., 2017; Tanisawa et al., 2020) Further, “sample and data storage”, (Pitsiladis et al., 2016; Vlahovich et al., 2017), “data sharing”, and “third-party access” (Webborn et al., 2015) have been highlighted by the reviewers. Also, the chance of incidental findings of mutations causing genetic disorders may have “severe psychological consequences such as depression or suicide” and consequences for “marriage, employment, life insurance, or reproductive choices”. (Wackerhage et al., 2009) Another ethical issue associated with genetic testing of athletes identified by the thematic analysis was “family relatedness” since the test results “may have direct health implications for other family members.” (Webborn et al., 2015)

#### 3.5.2 Theme: direct-to-consumer genetic testing companies

“Direct-to-consumer genetic testing companies” were identified by the thematic analysis and clustered as Threats. Several reviews mentioned that companies e-commerce directly to consumers sport-related genetic tests (McNamee et al., 2009; Guth and Roth, 2013; Breitbach et al., 2014; Webborn et al., 2015; Sarzynski et al., 2016; Vlahovich et al., 2017; Pickering et al., 2019; Naureen et al., 2020; Tanisawa et al., 2020; Griswold et al., 2021; Varillas-Delgado et al., 2022) although the AIS-Athlome consortium-FIMS joint statement from 2017 specified that “there is no current clinical application for genetic testing in the area of exercise prescription and injury prevention”. (Vlahovich et al., 2017) Often, commercial pressure would result in the “premature exploitation of data that have limited or no scientific bases given no or limited replication and validation”. (Tanisawa et al., 2020) Other companies would not “be disclosing what variants are being tested” (Sarzynski et al., 2016) and may use “misleading marketing claims”. (Vlahovich et al., 2017) Some authors argued that athletes and coaches might not be able to “understand the limitations and implications of genetic test results”. (Guth and Roth, 2013) Further, most of the companies “would not provide genetic counselling”, a central criticism mentioned by several reviewers with respect to direct-to-consumer genetic testing. (McNamee et al., 2009; Webborn et al., 2015; Vlahovich et al., 2017; Gray and Semsarian, 2020; Tanisawa et al., 2020) Finally, the “lack of universally accepted guidelines and legislation for direct-to-consumer genetic testing companies” has been highlighted by one reviewer. (Varillas-Delgado et al., 2022)

#### 3.5.3 Theme: gene doping

The last theme identified by the thematic analysis and highlighted by several reviewers was linked to “gene doping/editing”. (Sharp, 2008; Ostrander et al., 2009; Lippi et al., 2010; Vlahovich et al., 2017; Tanisawa et al., 2020; Varillas-Delgado et al., 2022) Gene doping/editing involves manipulating genetic material and regulating gene expression (e.g., increasing or decreasing the production of certain enzymes or proteins) to enhance athletic performance. (Cantelmo et al., 2020) One author stressed the possible side effects of gene editing. (Varillas-Delgado et al., 2022) Furthermore, the AIS-Athlome consortium-FIMS joint statement (2017) stated: “There is no role for gene-editing for the purposes of performance enhancement and all genetic manipulations are banned under the World Anti-Doping Agency”. (Vlahovich et al., 2017) According to Varillas Delgado et al. (2022) it is “unclear if there will ever be capacity to detect any type of gene modification by traditional laboratory techniques”. (Varillas-Delgado et al., 2022) In addition, “DNA testing and gene editing with embryos” was outlined as potential Threat. (Tanisawa et al., 2020) “Designing athletes with extraordinary athletic performance by using gene-editing technique would be a real threat in terms of keeping sport fair, clean and protecting athlete health” according to the sport and exercise genomics consensus update 2019. (Tanisawa et al., 2020)

## 4 Discussion

### 4.1 Summary of evidence

Many reviews (n = 25/45) of the present sSWOT described the potential of exercise genetics/genomics to contribute to precision exercise (Sharp, 2008; Lippi et al., 2010; Bouchard, 2011; Eynon et al., 2011; Guth and Roth, 2013; Pérusse et al., 2013; Wolfarth et al., 2014; Ahmetov and Fedotovskaya, 2015; Bouchard et al., 2015; Loos et al., 2015; Mattsson et al., 2016; Pitsiladis et al., 2016; Sarzynski et al., 2016; Vlahovich et al., 2017; Landen et al., 2019; Pickering et al., 2019; Pickering and Kiely, 2019; Gomes et al., 2020; Naureen et al., 2020; Tanisawa et al., 2020; Griswold et al., 2021; Sellami et al., 2021; Wang and Ashokan, 2021; Kim et al., 2022; Varillas-Delgado et al., 2022) but at the current state mainly aims to protect the health of athletes. (Tanisawa et al., 2020) Other applications (e.g., genotype scores, talent identification) are yet considered premature. (Eynon et al., 2011; Mattsson et al., 2016; Pickering et al., 2019; Naureen et al., 2020; Varillas-Delgado et al., 2022) Advances in technology together with large-scale multicenter studies, as well as the integration of multi-omics offer promising possibilities to uncover the complexity of exercise-related traits in the near future. (Bray, 2000; Brutsaert and Parra, 2006; Sharp, 2008; McNamee et al., 2009; Bouchard, 2011; Bouchard et al., 2011; Eynon et al., 2011; Hagberg et al., 2011; Pérusse et al., 2013; Wang et al., 2013; Breitbach et al., 2014; Wolfarth et al., 2014; Ahmetov and Fedotovskaya, 2015; Bouchard, 2015; Bouchard et al., 2015; Loos et al., 2015; Webborn et al., 2015; Gibson, 2016; Mattsson et al., 2016; Pitsiladis et al., 2016; Sarzynski et al., 2016; Wang et al., 2016; Yan et al., 2016; Moran and Pitsiladis, 2017; Vlahovich et al., 2017; Vellers et al., 2018; Landen et al., 2019; Pickering et al., 2019; Pickering and Kiely, 2019; Gomes et al., 2020; Tanisawa et al., 2020; Griswold et al., 2021; Sellami et al., 2021; Kim et al., 2022; Varillas-Delgado et al., 2022) Genetic profiling then may have the potential to optimize training and recovery strategies. (Sharp, 2008; Guth and Roth, 2013; Ahmetov and Fedotovskaya, 2015; Pitsiladis et al., 2016; Pickering et al., 2019; Naureen et al., 2020; Varillas-Delgado et al., 2022) The attainment of elite athlete status comprises a multifaceted interplay of intricate internal and external factors, such as adherence to a rigorous training process, optimal recovery protocols, optimized nutrition plans, etc. In light of this complexity, it appears highly improbable that genetic testing could ever serve as a viable means of accurately identifying prospective elite athletes. (Pickering et al., 2019; Tanisawa et al., 2020) At present, the general consensus among sport and exercise genetics researchers is that genetic tests have no role to play in talent identification. (Webborn et al., 2015)

The present sSWOT analysis also revealed several limitations related to exercise genetics/genomics, that were most frequently associated with methodological shortcomings especially linked to low quality studies. (Brutsaert and Parra, 2006; Ostrander et al., 2009; Wackerhage et al., 2009; Rankinen et al., 2010; Bouchard, 2011; Bouchard et al., 2011; Eynon et al., 2011; Hagberg et al., 2011; Roth et al., 2012; Guth and Roth, 2013; Wang et al., 2013; Breitbach et al., 2014; Ahmetov and Fedotovskaya, 2015; Bouchard, 2015; Bouchard et al., 2015; Loos et al., 2015; Webborn et al., 2015; Gibson, 2016; Mattsson et al., 2016; Pitsiladis et al., 2016; Wang et al., 2016; Moran and Pitsiladis, 2017; Vlahovich et al., 2017; Vellers et al., 2018; Landen et al., 2019; Pickering et al., 2019; Gomes et al., 2020; Naureen et al., 2020; Tanisawa et al., 2020; Griswold et al., 2021; Kim et al., 2022; Varillas-Delgado et al., 2022) These shortcomings can be overcome, at least partially, by using for instance consistent research protocols, (Vellers et al., 2018), appropriate study designs, (Bray, 2000; Brutsaert and Parra, 2006; Wang et al., 2013; Ahmetov and Fedotovskaya, 2015; Loos et al., 2015; Mattsson et al., 2016; Pitsiladis et al., 2016; Yan et al., 2016; Moran and Pitsiladis, 2017; Vlahovich et al., 2017), and correct statistics. (Rankinen et al., 2010; Bouchard, 2011; Hagberg et al., 2011; Roth et al., 2012; Wang et al., 2013; Wang et al., 2016) The difficulty of recruiting a large sample of (homogenous) elite athletes, however, will remain a challenge. Moreover, the relative high costs of funding, (Brutsaert and Parra, 2006; Wackerhage et al., 2009; Lippi et al., 2010; Rankinen et al., 2010; Hagberg et al., 2011; Roth et al., 2012; Gibson, 2016; Mattsson et al., 2016; Pitsiladis et al., 2016; Sarzynski et al., 2016), the presence of reporting bias, (Rankinen et al., 2010; Eynon et al., 2011; Wang et al., 2013; Gibson, 2016), and a rather limited research output of large-scale consortia were highlighted by some reviews. (Wang et al., 2013; Yan et al., 2016; Kim et al., 2022) In addition, (direct-to-consumer) genetic testing within the field of sport introduced many ethical questions and must be addressed to protect the privacy and health of athletes. (McNamee et al., 2009; Wackerhage et al., 2009; Guth and Roth, 2013; Breitbach et al., 2014; Webborn et al., 2015; Pitsiladis et al., 2016; Sarzynski et al., 2016; Wang et al., 2016; Vlahovich et al., 2017; Pickering et al., 2019; Gray and Semsarian, 2020; Naureen et al., 2020; Tanisawa et al., 2020; Griswold et al., 2021; Varillas-Delgado et al., 2022) Ultimately, the scourge of gene doping persists as a looming Threat to maintaining the sanctity of athletic competition, owing to the fact that the World Anti-Doping Agency has yet to endorse any standardized methodology for its detection. (Sharp, 2008; Ostrander et al., 2009; Lippi et al., 2010; Vlahovich et al., 2017; Tanisawa et al., 2020; Varillas-Delgado et al., 2022)

The most frequent themes comprised of “quality of studies” (Weakness) (31/45), “advances in technology” (Strength) (n = 31/45), “complexity of exercise-related traits” (Weakness) (n = 28/45), “empirical evidence” (Strength) (n = 25/45) as well as “precision exercise” (Opportunity) (n = 25/45) and “omics” (Opportunity) (n = 25/45). In contrast scarce themes included: “acceptance and accessibility” (Strengths) (n = 2/45), “research progress” (Weakness) (n = 2/45), “anti-doping detection” (Opportunity) (n = 2/45), “commercial use” (Opportunity) (n = 1/45), and “causality” (Weakness) (n = 1/45). Notably, the works of four publications have elucidated the sex-specific implications of exercise genetics, which we consider to be a significant yet inadequately represented subject matter within the present body of literature. (Mattsson et al., 2016; Vlahovich et al., 2017; Landen et al., 2019; Kim et al., 2022)

We also noticed a trend in time when extracting the data for the present sSWOT analysis: Reviews published around 2000 mainly described genetics, environment, and its interactions. (Bray, 2000; Brutsaert and Parra, 2006; Sharp, 2008) We then recognized a shift from reviews reporting on candidate gene studies (2000–2010) (Bray, 2000; Brutsaert and Parra, 2006; Ostrander et al., 2009; Rankinen et al., 2010) towards RNA expression profiling (2011) and genome-wide and whole exome association studies (2015). (Bouchard, 2011; Bouchard et al., 2011; Wang et al., 2013; Bouchard, 2015; Bouchard et al., 2015; Loos et al., 2015; Mattsson et al., 2016; Sarzynski et al., 2016; Wang et al., 2016; Vlahovich et al., 2017; Gomes et al., 2020) Genetic performance testing and associated ethical concerns were introduced around 2009 (Wackerhage et al., 2009; Guth and Roth, 2013; Breitbach et al., 2014; Webborn et al., 2015; Sarzynski et al., 2016) after disseminating the first genetic profile to predict human physical performance. (Williams and Folland, 2008) Precision approaches, multi-omics, and gene doping were more recently reported (from 2017 onwards) as more advanced technology developed. (Vellers et al., 2018; Landen et al., 2019; Pickering et al., 2019; Pickering and Kiely, 2019; Gomes et al., 2020; Naureen et al., 2020; Tanisawa et al., 2020; Griswold et al., 2021; Sellami et al., 2021; Kim et al., 2022; Varillas-Delgado et al., 2022)

This overview includes four consensus/consortia statements. (Webborn et al., 2015; Pitsiladis et al., 2016; Vlahovich et al., 2017; Tanisawa et al., 2020) First, the “Athlome Consortium project” (2016) comprises 15 international research groups and aims to uncover the omics basis of elite performance, training response, and predisposition to exercise-related injuries. (Pitsiladis et al., 2016) Next, two consensus statements on genetic testing, one from 2015 (Webborn et al., 2015) and one from 2017 (Vlahovich et al., 2017) concluded that “no child or young athlete should be exposed to direct-to-consumer genetic testing to define or alter training or for talent identification aimed at selecting gifted children or adolescents”, (Webborn et al., 2015), and “there is no current clinical application for genetic testing in the area of exercise prescription and injury prevention”. (Vlahovich et al., 2017) Finally, the “FIMS 2019 consensus statement” on sport and exercise genomics introduced rules of conduct for the ethical use of exercise genetics/genomics. (Tanisawa et al., 2020) In addition, in our opinion worth mentioning - although not included in the current overview - is a review of Lightfoot and others (2021) (Lightfoot et al., 2021) who provide recommendations regarding best-practice research standards and data analysis in the field of exercise genetics. We believe, conducting research in accordance with these recommendations can reduce some of the identified Threats and Weaknesses and allow further discoveries within exercise genetics/genomics in accordance with ethical principles.

### 4.2 Strengths and limitations

i) This is the first “overview of non-systematic reviews” related to exercise genetics/genomics including 45 reviews. By using a sSWOT approach, we used a systematic and efficient way to summarize the Strengths, Weaknesses, Opportunities, and Threats on the topic. ii) By searching two databases and reading the cross-references, we minimized the chance of missing relevant reviews. iii) Further, we employed a thematic analysis with three coding steps thereby increasing the internal validity of this overview. (Braun and Clarke, 2006; Nowell et al., 2017) iv) We strictly followed the study protocol and applied a checklist for conducting overviews of reviews. (Onishi and Furukawa, 2016) v) Finally, we used a systematic and transparent approach throughout the overview process in line with the European recommendations for research integrity. (Foundation and Academies, 2011)

This overview is not without limitations. i) As we included non-systematic reviews only, a quality assessment of the included reviews was not performed. Non-systematic reviews are prone to selection bias and may represent subjective opinions (e.g., positive or negative attitudes). (Yuan and Hunt, 2009) ii) We cannot rule out that themes have been missed in the current analysis. iii) One reviewer conducted the literature search and performed the data extraction. However, data extraction was performed twice within an 8-week wash out period thereby increasing the internal validity of the current report. iv) Finally, we excluded discipline- (e.g., endurance, strengths/power, team sports, injury, nutrition, psychological traits, etc.) and polymorphism-specific reviews. Interested readers are referred to the contemporary systematic review conducted by Ahmetov et al. (2022) which provides a comprehensive summary of the currently established genetic variants (n = 220) associated with athletic performance in a general context. (Ahmetov et al., 2022) Readers are further referred to following discipline specific reviews: endurance athletes, (Konopka et al., 2022), power/strengths athletes, (Maciejewska-Skrendo et al., 2019), combat sports, (Youn et al., 2021), cardiorespiratory fitness (i.e., the largest genome-wide association study to date with a non-athletic population), (Bye et al., 2020), injury, (Appel et al., 2021; Lim et al., 2021), nutrition, (Guest et al., 2019; Nieman, 2021), and psychology related reviews. (Silva et al., 2022) We also excluded reviews related to topics such as “doping”, “epigenetics”, and “omics” as we were interested in SWOTs linked to exercise genetics/genomics in general and not related to specific topics. The excluded reviews likely would influence the results of the applied analysis by increasing the number of identified themes such as “empirical evidence”, “omics and technology”, or “doping”.

## 5 Conclusion

We conducted a “systematic SWOT analysis of non-systematic reviews” including 45 reviews with the aim to identify the current SWOTs linked to exercise genetics/genomics. The thematic analysis identified five themes for Strengths and nine themes for Weaknesses. Six themes were linked to Opportunities, and three were clustered into Threats. Despite the complexity of exercise genetics/genomics, the present overview demonstrates that exercise genetics/genomics has future potential to assist athletes and coaches to enhance or maintain performance and health. The notion of precision exercise, whereby global large-scale consortia endeavor to establish correlations between genomics, multi-omics, and exercise-related traits, appears to hold considerable promise. This overview also highlights the need for further development in methodological and ethical guidance to minimize the identified Weaknesses and Threats. Furthermore, it seems justifiable to raise concerns regarding the relatively limited predictive capacity of genetic profiles. Nonetheless, the purpose of polygenic risk scores is not to make precise predictions about athletic performance, success, or health. Rather, genetic profiles should be utilized as an additional tool to enhance complex training methods. Noteworthy, the possession of an advantageous genotype does not guarantee the manifestation of athletic phenotypes, since a myriad of psychological, social, and environmental factors exert a substantial influence on athletic performance, and genetics only accounts for a fraction of the inter-individual variability observed. Nevertheless, the achievement of world-class levels will likely prove arduous without a favorable genetic profile.

## Data Availability

The original contributions presented in the study are included in the article/Supplementary Material, further inquiries can be directed to the corresponding author.
