# Supplementary material for: Genetics and athletic performance: a systematic SWOT analysis of non-systematic reviews
Source: Front Genet. 2023 Aug 9;14:1232987. doi: 10.3389/fgene.2023.1232987 (PMC10445150; doi:10.3389/fgene.2023.1232987)
Supplement: Supplementary file 1 [file Table1.docx]

Supplementary Material

# Supplementary 1. Checklist.

| Section/topic | # | Checklist item | Reported on page |
| --- | --- | --- | --- |
| Title and Abstract |  |  |  |
| Title | 1 | Identify the reports as an overview of (systematic) reviews, an umbrella review, or a meta-epidemiologic study | 1 |
| Structured summary | 2 | Provide a structured abstract | 2 |
| Introduction |  |  |  |
| Rationale | 3 | Specify the rationale for the overview of reviews in the context of an already-formed body of knowledge on the topic | 4 |
| Objectives | 4 | Describe a precise statement of questions | 4 |
| Methods |  |  |  |
| Protocol and registration | 5 | Report if an overview protocol was developed and if and where it can be obtained and provide registration information | 4 |
| Criteria for considering reviews for the overview | 6 | Describe review characteristics and report characteristics for eligibility criteria | 4, 5 |
| Information sources | 7 | State all information sources in the search and date last searched | 4, 5 |
| Search strategy | 8 | Specify full electronic search strategy including any limits used, such as language restriction | 4, 5  ESM 2 |
| Review selection | 9 | Provide the process for selecting reviews and its relevant details | 5 |
| Additional searches to identify other relevant primary studies | 10 | Report whether and why additional searches were conducted to identify other eligible primary studies | 4, 5 |
| Data extraction and management | 11 | State the processes of data extraction from included reviews and their relevant details | 5 |
| Data items | 12 | Specify all items overview authors sought (e.g., PICOS, methods, results, funding source) | 5 |
| Assessment of methodological quality of included reviews | 13 | Describe methods used for assessing methodological quality and quality of evidence and how this information was used for analyses | - |
| Data synthesis | 14 | Specify the methods of handling data and their details | 5 |
| Results |  |  |  |
| Review selection | 15 | Provide the details of review selection or a flow diagram of the overview process | Figure 1 |
| Review characteristics | 16 | Describe characteristics of each review (e.g., title, PICOS, number of studies and participants included, assessment of methodological quality of reviews, results of individual reviews) | ESM 3 |
| Assessment of methodological quality of included reviews | 17 | Report the results of assessment of methodological quality and quality of evidence of each included review | - |
| Syntheses of results | 18 | Summarize the main findings of the overview. If overview authors undertook data synthesis, present each summary measure with a confidence interval or a credible interval and measures of heterogeneity or inconsistency | 5-9  Table 1  ESM 5-8 |
| Discussion |  |  |  |
| Summary of evidence | 19 | Provide a concise summary of the main findings with the strength and shortcomings of evidence for each main outcome | 10, 11 |
| Limitations | 20 | Discuss limitations of the overview of review | 11, 12 |
| Conclusions | 21 | Present implications for practice and future research | 12 |
| Funding | 22 | Describe sources of funding for the overview of reviews | 13 |

# Supplementary 2. Full search strategy (date of last search: 08-01-2023).

|  | Medline | N results |
| --- | --- | --- |
| 1 | (athlete[Title/Abstract] OR athletic[Title/Abstract] OR exercise[Title/Abstract] OR sport[Title/Abstract])  AND (genetics[Title/Abstract] OR genomics[Title/Abstract])  Filters: Review  Sort by: Most Recent | 557 |

|  | Embase | N results |
| --- | --- | --- |
| 1 | athlete.ti. or athlete.ab. | 17971 |
| 2 | athletic.ti. or athletic.ab. | 20892 |
| 3 | exercise.ti. or exercise.ab. | 402884 |
| 4 | sport.ti. or sport.ab. | 48351 |
| 5 | 1 or 2 or 3 or 4 | 465464 |
| 6 | genetics.ti. or genetics.ab. | 159286 |
| 7 | genomics.ti. or genomics.ab. | 67905 |
| 8 | 6 or 7 | 218482 |
| 9 | 5 and 8 | 1546 |
| 10 | limit 9 to "review" | 427 |

# Supplementary 3. Excluded studies and reason for exclusion (n=44).

| First author | Year | Title | Reason for exclusion |
| --- | --- | --- | --- |
| Eynon^83^ | 2013 | Genes for elite power and sprint performance: ACTN3 leads the way | discipline |
| Maffulli^84^ | 2013 | The genetics of sports injuries and athletic performance | discipline |
| Tucker^85^ | 2013 | The genetic basis for elite running performance | discipline |
| Vancini^86^ | 2014 | Genetic aspects of athletic performance: the African runners phenomenon | discipline |
| Heffernan^87^ | 2015 | Genomics in rugby union: A review and future prospects | discipline |
| Lundby^88^ | 2017 | Biology of VO(2) max: looking under the physiology lamp | discipline |
| Costello^90^ | 2018 | Sport related concussion - Potential for biomarkers to improve acute management | discipline |
| Southward^89^ | 2018 | The Role of Genetics in Moderating the Inter-Individual Differences in the Ergogenicity of Caffeine | discipline |
| Brazier^91^ | 2019 | Tendon and Ligament Injuries in Elite Rugby: The Potential Genetic Influence | discipline |
| Herbert^82^ | 2019 | The interactions of physical activity, exercise and genetics and their associations with bone mineral density: implications for injury risk in elite athletes | discipline |
| Antrobus^93^ | 2021 | Genetic Factors That Could Affect Concussion Risk in Elite Rugby | discipline |
| Barreto^94^ | 2021 | Novel insights on caffeine supplementation, CYP1A2 genotype, physiological responses and exercise performance | discipline |
| Mareddy^95^ | 2022 | Exercise in the Genetic Arrhythmia Syndromes - A Review | discipline |
| Meyler^96^ | 2021 | Biological and methodological factors affecting V̇O2max response variability to endurance training and the influence of exercise intensity prescription | discipline |
| Cabrera-Serrano^97^ | 2022 | Recent advances in our understanding of genetic rhabdomyolysis | discipline |
| Ehlert^15^ | 2013 | Epigenetics in sports | omics/epigenetics |
| Bassini^98^ | 2014 | Sportomics: building a new concept in metabolic studies and exercise science | omics |
| Kennedy^99^ | 2018 | Metabolomics in the clinic: A review of the shared and unique features of untargeted metabolomics for clinical research and clinical testing | omics |
| Hill^100^ | 2019 | Bioenergetics and translational metabolism: implications for genetics, physiology and precision medicine | omics |
| Polli^101^ | 2019 | When Environment Meets Genetics: A Clinical Review of the Epigenetics of Pain, Psychological Factors, and Physical Activity | omics/epigenetics |
| Hall^102^ | 2020 | The Prospective Study of Epigenetic Regulatory Profiles in Sport and Exercise Monitored Through Chromosome Conformation Signatures | omics/epigenetics |
| Kelly^103^ | 2020 | Metabolomics, physical activity, exercise and health: A review of the current evidence | omics |
| Malsagova^104^ | 2021 | Molecular Portrait of an Athlete | omics |
| Nieman^105^ | 2021 | Multiomics Approach to Precision Sports Nutrition: Limits, Challenges, and Possibilities | omics |
| Schneider^106^ | 2012 | Human genetic variation: new challenges and opportunities for doping control | doping |
| Fischetto^107^ | 2013 | From gene engineering to gene modulation and manipulation: can we prevent or detect gene doping in sports? | doping |
| Unal^108^ | 2004 | Gene doping in sports | doping |
| Gaffney^109^ | 2007 | Gene doping: a review of performance-enhancing genetics | doping |
| Zhang^110^ | 2019 | Genetic Factors Associated With Human Physical Activity: Are Your Genes Too Tight To Prevent You Exercising? | heritability |
| Gordon^111^ | 2005 | The genetics of muscle atrophy and growth: the impact and implications of polymorphisms in animals and humans | polymorphism |
| Sarpeshkar^112^ | 2010 | [Adrenergic-β 2 receptor polymorphism and athletic performance](https://www.nature.com/articles/jhg201042) | polymorphism |
| Angelopoulos^113^ | 2011 | Genetics of the adaptation to exercise | polymorphism |
| Collins^114^ | 2016 | Genetics of Musculoskeletal Exercise-Related Phenotypes | polymorphism |
| Venezia^115^ | 2016 | Recent Research in the Genetics of Exercise Training Adaptation | polymorphism |
| Pickering^116^ | 2017 | ACTN3: More than Just a Gene for Speed | polymorphism |
| Del Coso^8^ | 2019 | More than a 'speed gene': ACTN3 R577X genotype, trainability, muscle damage, and the risk for injuries | polymorphism |
| van de Vegte^117^ | 2019 | Genetics and the heart rate response to exercise | polymorphism |
| Rankinen^118^ | 2006 | The human gene map for performance and health-related fitness phenotypes: the 2005 update | systematic review |
| Alvarez-Romero^119^ | 2021 | Mapping Robust Genetic Variants Associated with Exercise Responses | systematic review |
| Balberova^120^ | 2021 | Candidate Genes of Regulation of Skeletal Muscle Energy Metabolism in Athletes | systematic review |
| Ginevičienė^18^ | 2022 | Perspectives in Sports Genomics | systematic review |
| Ahmetov^21^ | 2022 | Advances in sports genomics | systematic review |
| Konopka^121^ | 2022 | Genetics of long‐distance runners and road cyclists—A systematic review with meta‐analysis | systematic review |
| Mohan^122^ | 2022 | A Mini Review on Sports Genetics Researches in India: Where We Stand Now | systematic review |

# Supplementary 4. Study characteristics of the 45 reviews presenting the main themes clustered into Strengths, Weaknesses, Opportunities, and Threats.

| First author | Year | Title | Strengths | Weaknesses | Opportunity | Threat | |
| --- | --- | --- | --- | --- | --- | --- | --- |
|  |  |  | **Thematic phrases (Themes)** | | | | |
| Bray^43^ | 2000 | Genomics, genes, and environmental interaction: the role of exercise | -technology | -complex trait | -omics/ technology | | - |
| Brutsaert^44^ | 2006 | What makes a champion? Explaining variation in human athletic performance | -evidence  -growing field  -technology | -complex trait  -costs  -quality | -omics/ technology | | - |
| Sharp^45^ | 2008 | The human genome and sport, including epigenetics and athleticogenomics: a brief look at a rapidly changing field | -technology | - | -precision exercise/ profiling  -screening/ therapy | | -doping |
| McNamee^46^ | 2009 | Genetic testing and sports medicine ethics | -technology | - | -screening/ therapy | | -DTC-GT  -ethics |
| Ostrander^47^ | 2009 | Genetics of athletic performance | -evidence | -quality | - | | -doping |
| Wackerhage^48^ | 2009 | Genetic research and testing in sport and exercise science: a review of the issues | -evidence | -complex trait  -costs  -quality | -screening/ therapy | | -ethics |
| Lippi^49^ | 2010 | Genetics and sports | -accessibility  -evidence  -technology | -complex trait  -costs | -anti-doping  -precision exercise/ profiling  -screening/ therapy | | -doping |
| Rankinen^50^ | 2010 | Advances in exercise, fitness, and performance genomics | - | -generalizability  -reporting bias  -quality | - | | - |
| Bouchard^51^ | 2011 | Overcoming barriers to progress in exercise genomics | -evidence  -technology | -complex trait  -quality | -multicentre studies  -omics/ technology  -precision exercise/ profiling  -screening/ therapy | |  |
| Bouchard^52^ | 2011 | Genomics and genetics in the biology of adaptation to exercise | -technology | -complex trait  -generalizability  -quality | -omics/ technology | | - |
| Eynon^53^ | 2011 | Genes and elite athletes: a roadmap for future research | -evidence  -technology | -complex trait  -generalizability  -genotype scores  -invasive methods  -reporting bias  -quality | -multicentre studies  -omics/ technology  -precision exercise/ profiling | | - |
| Hagberg^54^ | 2011 | Advances in exercise, fitness, and performance genomics in 2010 | - | -costs  -quality | -multicentre studies | | - |
| Roth^55^ | 2012 | Advances in exercise, fitness, and performance genomics in 2011 | - | -complex trait  -costs  -quality | - | | - |
| Guth^56^ | 2013 | Genetic influence on athletic performance | -evidence | -complex trait  -quality | -precision exercise/ profiling | | -DTC-GT  -ethics |
| Pérusse^57^ | 2013 | Advances in exercise, fitness, and performance genomics in 2012 | -technology | - | -omics/ technology  -precision exercise/ profiling  -screening/ therapy | | - |
| Wang^58^ | 2013 | Genomics of elite sporting performance: what little we know and necessary advances | -consortia  -evidence  -technology | -complex trait  -genotype scores  -reporting bias  -research progress  -quality | -multicentre studies  -omics/ technology | | - |
| Breitbach^59^ | 2014 | Conventional and genetic talent identification in sports: will recent developments trace talent? | -evidence  -technology | -complex trait  -genotype scores  -quality | -screening/ therapy | | -DTC-GT  -ethics |
| Wolfarth^60^ | 2014 | Advances in exercise, fitness, and performance genomics in 2013 | -growing field  -technology | -complex trait | -precision exercise/ profiling  -omics/ technology | | - |
| Ahmetov^19^ | 2015 | Current Progress in Sports Genomics | -evidence  -growing field  -technology | -complex trait  -generalizability -quality | -multicentre studies  -precision exercise/ profiling  -omics/ technology | | - |
| Bouchard^17^ | 2015 | Exercise genomics--a paradigm shift is needed: a commentary | -technology | -complex trait -quality | -multicentre studies  -omics/ technology  -screening/ therapy | | - |
| Bouchard^61^ | 2015 | Personalized preventive medicine: genetics and the response to regular exercise in preventive interventions | -evidence  -technology | - | -omics/ technology  -precision exercise/ profiling | | - |
| Loos^62^ | 2015 | Advances in exercise, fitness, and performance genomics in 2014 | -growing field  -technology | -complex trait  -costs  -quality | -multicentre studies  -precision exercise/ profiling | | - |
| Webborn^63^ | 2015 | Direct-to-consumer genetic testing for predicting sports performance and talent identification: Consensus statement | -technology | -generalizability  -genotype scores  -quality | - | | -DTC-GT  -ethics |
| Gibson^64^ | 2016 | Core Concepts in Human Genetics: Understanding the Complex Phenotype of Sport Performance and Susceptibility to Sport Injury | -growing field  -technology | -complex trait  -costs  -invasive methods  -reporting bias  -quality | -omics/ technology | | - |
| Mattsson^65^ | 2016 | Sports genetics moving forward: lessons learned from medical research | -technology | -causality  -complex trait  -generalizability  -genotype scores  -invasive methods  -quality | -multicentre studies  -omics/ technology  -precision exercise/ profiling | | - |
| Pitsidalis^66^ | 2016 | Athlome Project Consortium: a concerted effort to discover genomic and other "omic" markers of athletic performance | -consortia | -quality | -omics/ technology  -precision exercise/ profiling | | -ethics |
| Sarzynski^67^ | 2016 | Advances in Exercise, Fitness, and Performance Genomics in 2015 | -technology | -costs | -omics/ technology  -precision exercise/ profiling | | -DTC-GT  -ethics |
| Wang^68^ | 2016 | The Future of Genomic Research in Athletic Performance and Adaptation to Training | -consortia  -evidence  -technology | -complex trait  -generalizability  -quality | -multicentre studies  -omics/ technology | | -ethics |
| Yan^69^ | 2016 | Nature versus Nurture in Determining Athletic Ability | -evidence  -technology | -complex trait  -research progress | - | | - |
| Moran^70^ | 2017 | Tour de France Champions born or made: where do we take the genetics of performance? | -evidence  -technology | -complex trait  -quality | -multicentre studies  -omics/ technology | | - |
| Vlahovich^26^ | 2017 | Genetic testing for exercise prescription and injury prevention: AIS-Athlome consortium-FIMS joint statement | -consortia  -growing field  -technology | -generalizability  -quality | -multicentre studies  -precision exercise/ profiling  -screening/ therapy | | -doping  -DTC-GT  -ethics |
| Vellers^71^ | 2018 | Inter-individual variation in adaptations to endurance and resistance exercise training: genetic approaches towards understanding a complex phenotype | -evidence  -technology | -complex trait  -quality | -multicentre studies | | - |
| Landen^72^ | 2019 | Genetic and epigenetic sex-specific adaptations to endurance exercise | -evidence  -technology | -complex trait  -generalizability  -quality | -multicentre studies  -omics/ technology  -precision exercise/ profiling | | - |
| Pickering^73^ | 2019 | The Development of a Personalised Training Framework: Implementation of Emerging Technologies for Performance | -acceptance  -growing field  -technology | -complex trait  -invasive methods | -omics/ technology  -precision exercise/ profiling | | - |
| Pickering^74^ | 2019 | Can Genetic Testing Identify Talent for Sport? | -evidence  -technology | -complex trait  -generalizability  -genotype scores  -quality | -precision exercise/ profiling  -screening/ therapy | | -DTC-GT  -ethics |
| Gomes^75^ | 2020 | Omics and the molecular exercise physiology | -consortia  -evidence  -technology | -complex trait  -quality | -multicentre studies  -omics/ technology  -precision exercise/ profiling | | - |
| Gray^76^ | 2020 | Utility of genetic testing in athletes for cardiac diseases | - | - | - | | -ethics |
| Naureen^77^ | 2020 | Genetic test for the personalization of sport training | -evidence | -generalizability  -genotype scores  -quality | -precision exercise/ profiling | | -DTC-GT  -ethics |
| Tanisawa^9^ | 2020 | Sport and exercise genomics: the FIMS 2019 consensus statement update | -consortia  -evidence  -technology | -complex trait  -quality | -commercial use  -omics/ technology  -precision exercise/ profiling  -screening/ therapy | | -doping  -DTC-GT  -ethics |
| Griswold^78^ | 2021 | Using Genomic Techniques in Sports and Exercise Science: Current Status and Future Opportunities | -consortia  -evidence  -technology | -complex trait  -generalizability  -quality | -multicentre studies  -omics/ technology  -precision exercise/ profiling  -screening/ therapy | | -DTC-GT |
| Sellami^16^ | 2021 | Molecular Big Data in Sports Sciences: State-of-Art and Future Prospects of OMICS-Based Sports Sciences | -consortia | - | -multicentre studies  -omics/ technology  -precision exercise/ profiling | | - |
| Wang^79^ | 2021 | Physical Exercise: An Overview of Benefits From Psychological Level to Genetics and Beyond | -evidence | - | -precision exercise/ profiling | | - |
| Kim^80^ | 2022 | The genetics of human performance | -evidence | -generalizability  -quality | -multicentre studies  -omics/ technology  -precision exercise/ profiling | | - |
| Varillas-Delgado^81^ | 2022 | Genetics and sports performance: the present and future in the identification of talent for sports based on DNA testing | -evidence  -growing field | -complex trait  -genotype scores  -quality | -anti-doping  -multicentre studies  -omics/ technology  -precision exercise/ profiling | | -doping  -DTC-GT |
| Vicelli^82^ | 2022 | The non-modifiable factors age, gender, and genetics influence resistance exercise | -evidence | - | - | | - |

DTC-GT=direct-to-consumer genetic testing.

# Supplementary 5. Content analysis – Strengths.

| First author | Year | Title | Initial Coding (citations from the articles, chronological order in text may be changed) | Focused  Coding | Theoretical  Coding |
| --- | --- | --- | --- | --- | --- |
| Bray^43^ | 2000 | Genomics, genes, and environmental interaction: the role of exercise | As new technologies presently being developed for differential gene expression research, such as chip-based microarrays, become more widely used, an increasing number of genes whose expression is modified by exercise will be identified. | -technology | Strengths |
| Brutsaert^44^ | 2006 | What makes a champion? Explaining variation in human athletic performance | One frequently cited study is that by Bouchard and colleagues revealing significant familial aggregation (h2 near 50%) for VO2 max (maximal oxygen uptake) and VO2 max trainability for individuals in the sedentary state, after controlling for age, sex, body mass, and body composition.  Candidate gene studies are relatively new and have proliferated greatly since the early 1990s. There has been a recent explosion of interest in the genetic basis of human athletic performance paralleling the development of new and accessible genotyping and DNA sequencing technologies. | -evidence  -growing field  -technology | Strengths |
| Sharp^45^ | 2008 | The human genome and sport, including epigenetics and athleticogenomics: a brief look at a rapidly changing field | Sanger DNA sequencing technology reads about 70,000 base-pairs per hour, but picolitre-sized chemical reactor technology, in a casing the size of a microwave oven, will soon be 100 times faster, at about six million base-pairs per hour. | -technology | Strengths |
| McNamee^46^ | 2009 | Genetic testing and sports medicine ethics | Given that genomic medicine and technology are advancing so rapidly, it is worth considering their potential ethical impact in advance of actual medical applications in athletics. | -technology | Strengths |
| Ostrander^47^ | 2009 | Genetics of athletic performance | It is now believed that over 200 PEPs (performance enhancing polymorphisms) exist. | -evidence | Strengths |
| Wackerhage^48^ | 2009 | Genetic research and testing in sport and exercise science: a review of the issues | For example, the heritability of maximal oxygen uptake (VO2max) may be as high as 50% and the heritability of the trainability of VO2max has been estimated to be 47%. | -evidence | Strengths |
| Lippi^49^ | 2010 | Genetics and sports | Although we all would agree that genetic testing is not aimed to replace the findings on the athletic field, under the most appropriate circumstances it might be less invasive, less expensive and more accurate than conventional in vivo or in vitro analyses.  Physical fitness has also a strong genetic component, up to 50%.  Advances in genetic knowledge, high-throughput genotyping technologies, statistical analysis algorithms and a flood of data on human genetic variation from the Human Genome and HapMap projects have made GWA (genome wide association) studies technically feasible, allowing to identify genetic variants that are associated with complex human diseases. | -accessibility  -evidence  -technology | Strengths |
| Rankinen^50^ | 2010 | Advances in exercise, fitness, and performance genomics | - | - | - |
| Bouchard^51^ | 2011 | Overcoming barriers to progress in exercise genomics | Collectively, these 16 SNPs (single nucleotide polymorphisms) explained 45% of the variance in ṾO2max training response, which is very close to the maximal heritability estimate of 47% reported previously in the HERITAGE Family Study.  The extraordinary progress made in the past decade on the sequence and information content of the human genome and the genomes of commonly used animal models, on high-throughput technologies for genotyping and sequencing whole genomes, and on transcriptomics has generated a lot of enthusiasm regarding our ability to elucidate the genetic, genomic, and molecular basis of human variation in health and disease. | -evidence  -technology | Strengths |
| Bouchard^52^ | 2011 | Genomics and genetics in the biology of adaptation to exercise | With the extraordinary progress made in sequencing technologies, it has become feasible to sequence long stretches of DNA in many samples. | -technology | Strengths |
| Eynon^53^ | 2011 | Genes and elite athletes: a roadmap for future research | For instance, ∼66% of the variance in athlete status is explained by additive genetic factors, with the remaining variance being attributable to non-shared environmental factors.  Genome-wide scan linkage or genome-wide association studies allow the analysis of polymorphic markers of the whole genome combined with informatics and robust statistics to link genetic markers to physiological phenotypes. | -evidence  -technology | Strengths |
| Hagberg^54^ | 2011 | Advances in exercise, fitness, and performance genomics in 2010 | - | - | - |
| Roth^55^ | 2012 | Advances in exercise, fitness, and performance genomics in 2011 | - | - | - |
| Guth^56^ | 2013 | Genetic influence on athletic performance | As of 2009, more than 200 genetic variants had been associated with physical performance, with more than 20 variants being associated with elite athlete status.  The heritability of athletic status (regardless of sport) is estimated to be 66%. | -evidence | Strengths |
| Pérusse^57^ | 2013 | Advances in exercise, fitness, and performance genomics in 2012 | Even though the increasing reliance on unbiased genome-wide screens is clearly a strength, we again note that the vast majority of exercise genomics studies (defined in a broad sense), including many that have not been reviewed herein, reported in 2012 are observational. | -technology | Strengths |
| Wang^58^ | 2013 | Genomics of elite sporting performance: what little we know and necessary advances | New approaches involving large, well-funded consortia and utilising well-phenotyped large cohorts and genome-wide technologies will be necessary for meaningful progress to be made.  Over 200 SNPs associated with physical-performance traits, and over 20 SNPs associated with elite athletic status, have been reported in the literature and summarised on a yearly basis in the ‘The Human Gene Map for Performance and Health-related Fitness Phenotypes’ until 2009.  The hypothesis-free GWAS (genome wide association study) design is the most popular of the current widespread approaches as it allows one to (1) detect smaller gene effects by narrowing down the genomic target region precisely with new chips; (2) maximise the amount of variation captured per SNP with a fixed set of markers and (3) reduce genotyping costs, which make this approach attractive. | -consortia  -evidence  -technology | Strengths |
| Breitbach^59^ | 2014 | Conventional and genetic talent identification in sports: will recent developments trace talent? | Heritability studies in performance-related issues found that most of the traits accounted for 15–65 % of the total variance, indicating small to moderate genetic influences.  Since the start of early association studies, many technical efforts have been achieved in the field of genotyping approaches, initiating an even broader field of possibilities but also pointing out further barriers. | -evidence  -technology | Strengths |
| Wolfarth^60^ | 2014 | Advances in exercise, fitness, and performance genomics in 2013 | It is obvious to us, and it should be to those who have been following the yearly instalments of the reviews, that the field has grown in sophistication and that the science has become stronger.  What is becoming increasingly clear is that exercise scientists who have an interest in exercise genomics questions are adopting new emerging technologies at a faster pace compared with prior decades. | -growing field  -technology | Strengths |
| Ahmetov^19^ | 2015 | Current Progress in Sports Genomics | Accordingly, athlete status is a heritable trait: on average 66% (depends on sporting discipline) of the variance in athlete status is explained by additive genetic factors.  As the figure shows, most of these polymorphisms (70%) were discovered in the last 5 years (2010–2014), indicating a growing interest in the field of sports genomics.  The next decade will be an exciting period for sports genomics, as we apply the new DNA technologies (whole-genome sequencing, GWAS, epigenomic, transcriptomic, proteomic profiling, etc.) and bioinformatics to further dissect and analyze the genetic effects on human physical ability. | -evidence  -growing field  -technology | Strengths |
| Bouchard^17^ | 2015 | Exercise genomics--a paradigm shift is needed: a commentary | Over the last decade, with the advent of technological advances allowing to genotype millions of common single nucleotide polymorphisms (SNPs) in each individual of a study, the investigation of the contribution of common variants to human characteristics and disease outcomes has grown exponentially. | -technology | Strengths |
| Bouchard^61^ | 2015 | Personalized preventive medicine: genetics and the response to regular exercise in preventive interventions | A model-fitting analytical procedure yielded a maximal heritability estimate of 47% for VO2Max response level.  The completion of the Human Genome Project and the subsequent expansion of genetic studies to genome-wide association studies (GWASs) and whole genome and whole exome sequencing have led to a new era in genetics. | -evidence  -technology | Strengths |
| Loos^62^ | 2015 | Advances in exercise, fitness, and performance genomics in 2014 | It is obvious to us, and it should be to those who have been following the yearly instalments of the reviews, that the field has grown in sophistication and that the science has become stronger.  The results reported for the first type of studies, using GWAS-identified SNPs, tend to be more robust and often replicate findings from preceding years. | -growing field  -technology | Strengths |
| Webborn^63^ | 2015 | Direct-to-consumer genetic testing for predicting sports performance and talent identification: Consensus statement | The pace of advance in sequencing and genotyping technology has far exceeded the pace of change in related regulation. | -technology | Strengths |
| Gibson^64^ | 2016 | Core Concepts in Human Genetics: Understanding the Complex Phenotype of Sport Performance and Susceptibility to Sport Injury | The rapid drop in costs for SNP genotyping and DNA sequencing has spurred an equally rapid growth in studies that attempt to correlate DNA sequence variants with variation in measurable physical characteristic.  Novel sequencing technologies on the horizon for sport science include whole-exome sequencing, whole-genome sequencing, transcriptome analysis and epigenetic profiling. | -growing field  -technology | Strengths |
| Mattsson^65^ | 2016 | Sports genetics moving forward: lessons learned from medical research | Since then, we have seen dramatic advances in technology and reduction in cost. | -technology | Strengths |
| Pitsidalis^66^ | 2016 | Athlome Project Consortium: a concerted effort to discover genomic and other "omic" markers of athletic performance | The Athlome Project aims to collectively study the genotype and phenotype data currently available on elite athletes, in adaptation to exercise training (in both human and animal models) and on exercise-related musculoskeletal injuries from individual studies and from consortia worldwide. | -consortia | Strengths |
| Sarzynski^67^ | 2016 | Advances in Exercise, Fitness, and Performance Genomics in 2015 | In summary, the exercise genomics field is poised for substantial progress in the next decade, particularly with the advent of the NIH Common Fund MoTrPAC program and the continuing development and decreasing cost of high- throughput technologies. | -technology | Strengths |
| Wang^68^ | 2016 | The Future of Genomic Research in Athletic Performance and Adaptation to Training | At the end of the symposium, the participants agreed to launch a large collaborative initiative (named ‘the Athlome Project Consortium’, www.athlomeconsortium.org) to share and bring together resources for future investigations.  For example, the heritability of athletic status, trainability, and exercise behaviour is estimated to be 66, 47, and 62%, respectively. As of 2008, over 200 genes were reported to be significantly associated with human physical performance.  Hypothesis-free genome-wide approaches will in the future provide more comprehensive coverage and in-depth understanding of the biology underlying sports-related traits and related genetic mechanisms. | -consortia  -evidence  -technology | Strengths |
| Yan^69^ | 2016 | Nature versus Nurture in Determining Athletic Ability | Studies on twins (involving both identical and non-identical twins) and family members, which implicate genetics as a significant factor pertaining to athletic performance and adaptation to exercise training (even after adjusting for environmental factors), have shown that 30–80% of the variation in various performance traits (phenotypes) can be attributed to genetics. Using predominately hypothesis-driven case-control candidate gene association studies, over 200 polymorphisms associated with exercise-related traits and over 20 polymorphisms associated with elite athletic status have been reported in the literature and are summarized on a yearly basis.  As opposed to the hypothesis-driven approach of looking at one or a few targeted polymorphisms and assessing their association with athletic ability, an ‘unbiased’, whole-genome sequencing approach has recently come into use. | -evidence  -technology | Strengths |
| Moran^70^ | 2017 | Tour de France Champions born or made: where do we take the genetics of performance? | Due to the relatedness of the family members, they were able to estimate that approximately 50% of the variance in VO2max could be explained by genetic variation (whether measured at baseline, i.e., natural talent, or in response to training). This implies that genetic variation is at least as important to performance as everything else put together, i.e., lifestyle, diet, training, facilities, etc.  However, with the ever-decreasing cost and increasing speed of next generation DNA sequencing (NGS), it is now feasible to perform whole genome DNA sequencing on large cohorts of individuals thus identifying unknown rare variants and allowing testing of their associations with quantitative phenotypes. | -evidence  -technology | Strengths |
| Vlahovich^26^ | 2017 | Genetic testing for exercise prescription and injury prevention: AIS-Athlome consortium-FIMS joint statement | The Athlome Project Consortium was established in 2015 to collectively study the limited genotype and phenotype data available in elite athletes, as well as focusing on adaptation to exercise training and exercise-related musculoskeletal injuries.  The rapid development of genetic and genomic techniques has led to an increase in interest in the genetics of physical activity and sport.  International collaborations and utilising high throughput sequencing technologies to identify genes that contribute to exercise response. | -consortia  -growing field  -technology | Strengths |
| Vellers^71^ | 2018 | Inter-individual variation in adaptations to endurance and resistance exercise training: genetic approaches towards understanding a complex phenotype | Genetic background has been determined as a strong contributor to inter-individual variation in such measures of endurance and resistance training-induced adaptations.  Recent advances in technologies, including Next-generation Sequencing, have enabled complete and ultra-deep sequencing of mitochondrial DNA in animal and human tissues that contain mitochondria. | -evidence  -technology | Strengths |
| Landen^72^ | 2019 | Genetic and epigenetic sex-specific adaptations to endurance exercise | The heritability of VO2max, a strong indicator of endurance performance, is estimated to be between ~22–57%, meaning that ~22–57% of the variability in VO2max observed in a population can be attributed to genetic variation.  Genome-wide association studies (GWAS) have emerged as a more effective way to determine the contribution of SNPs to a specific trait or phenotype. | -evidence  -technology | Strengths |
| Pickering^73^ | 2019 | The Development of a Personalised Training Framework: Implementation of Emerging Technologies for Performance | High-level athletes and support staff appear interested in utilising genetic information to enhance their performance, with recent surveys suggesting that ~10% of athletes have done so already, with many interested in doing so in the future.  Such a finding could be very useful within elite sport because muscle biopsy testing is highly invasive, limiting its use, while genetic testing is non-invasive.  Exercise genetics research has grown over the last thirty years, from the seminal HERITAGE study to Genome-Wide Association Study approaches, and, most recently, the development of Total Genotype Scores to utilise genetic information within the contexts of training programme design. | -acceptance  -growing field  -technology | Strengths |
| Pickering^74^ | 2019 | Can Genetic Testing Identify Talent for Sport? | 155 genetic markers have been tentatively linked with elite athlete status.  Recent advances in genetic technology have allowed for greater exploration of the genetic underpinnings of elite performance, leading to the identification of single nucleotide polymorphisms (SNPs) and other genetic variants with the potential to influence sports performance, either directly or indirectly. | -evidence  -technology | Strengths |
| Gomes^75^ | 2020 | Omics and the molecular exercise physiology | Large consortia were created to achieve ambitious goals.  This new field was interested in identifying the genetic link to endurance performance, suggesting that genetics is a significant component of aerobic capacity. This hypothesis was supported by studies in animal models [and humans, mainly represented by the HERITAGE Family Study, which indicated that genetic heritability was responsible for almost 50% of VO2max.  The year 2001 marked the beginning of a revolution in biological research due to the completion of the sequencing of the human genome. The development of cost-effective genotyping technologies, such as DNA microarrays, allowed the investigation of small nucleotide polymorphisms (SNPs). | -consortia  -evidence  -technology | Strengths |
| Gray^76^ | 2020 | Utility of genetic testing in athletes for cardiac diseases | - | - | - |
| Naureen^77^ | 2020 | Genetic test for the personalization of sport training | During the last two decades, several studies have provided compelling evidence that both endurance and power performances are influenced by genetic factors that collectively are called performance-enhancing gene polymorphisms (PEPs). Surprisingly, PEPs are common in general population and more than 200 PEPs have been reported so far. | -evidence | Strengths |
| Tanisawa^9^ | 2020 | Sport and exercise genomics: the FIMS 2019 consensus statement update | Progress towards such a significant development in the field of sport and exercise genomics will require a paradigm shift in line with recent recommendations for international collaborations such as the Athlome Project Consortium (see www.athlomeconsortium.org) which was launched in 2015 for the advancement of ‘omics’ in exercise sciences and medicine.  Previous twin and familial studies suggest that there is moderate heritability of ‘sport and exercise-related traits’ (eg, athletic performance, response to exercise training and fitness level).  Recent advances in DNA sequencing technology enable the analysis of a large number of genome. | -consortia  -evidence  -technology | Strengths |
| Griswold^78^ | 2021 | Using Genomic Techniques in Sports and Exercise Science: Current Status and Future Opportunities | Notably, there are ongoing efforts in existing consortia including athletic performance collections.  In the area of sports medicine, these study designs have supported a role for genetics in traits related to athletic performance, such as VO2max and response  to resistance training, with heritability estimates ranging from 0.4 to 0.7, suggesting that 40% to 70% of the variability in VO2max across the population is explained by genetic factors.  It propelled the development of new ideas and technologies which dramatically lowered the cost and time required for assaying DNA variation across the genome via high throughput genotyping arrays, such as in genome-wide association studies and then by next generation massively parallel sequencing of entire exome and genomes of hundreds and thousands of individuals. Moreover, technological improvements have allowed sequencing of whole genomes, rather than typing of only specific markers, expanding studies into rare variant discovery. | -consortia  -evidence  -technology | Strengths |
| Sellami^16^ | 2021 | Molecular Big Data in Sports Sciences: State-of-Art and Future Prospects of OMICS-Based Sports Sciences | Several projects and trials (like ELITE, GAMES, Gene SMART, GENESIS, and POWERGENE) are aimed at discovering genomics- and post-genomics-based biomarkers with an adequate predictive power, statistical robustness, and interpretability of discoveries in terms of replicable, physiologically meaningful, and relevant patterns. | -consortia | Strengths |
| Wang^79^ | 2021 | Physical Exercise: An Overview of Benefits From Psychological Level to Genetics and Beyond | Studies have also identified heritability to affect VO2 max response to exercise training by 47%. | -evidence | Strengths |
| Kim^80^ | 2022 | The genetics of human performance | It is largely accepted that an individual’s maximal performance is strongly heritable. | -evidence | Strengths |
| Varillas-Delgado^81^ | 2022 | Genetics and sports performance: the present and future in the identification of talent for sports based on DNA testing | Despite a relatively strong influence of heritability on the probability of becoming an elite athlete (> 70%, depending on the sports discipline) or on the values of phenotypes typically associated with performance.  Research focused on ascertaining the effect of genetics on exercise traits has been progressively growing in the last few years. | -evidence  -growing field | Strengths |
| Vicelli^82^ | 2022 | The non-modifiable factors age, gender, and genetics influence resistance exercise | They reported transmissibility for muscle mass to be greater than 90%. | -evidence | Strengths |

# Supplementary 6. Content analysis – Weaknesses.

| First author | Year | Title | Initial Coding (citations from the articles, chronological order in text may be changed) | Focused  Coding | Theoretical  Coding |
| --- | --- | --- | --- | --- | --- |
| Bray^43^ | 2000 | Genomics, genes, and environmental interaction: the role of exercise | One explanation for the lack of findings may be that detection of the effects of any one polymorphism on a complex trait, such as metabolic rate, for example, may be dependent on the context in which the variant is being analyzed (e.g., exercisers vs. nonexercisers). Traits such as energy metabolism, VO2 max, blood pressure, blood lipid levels, body fat, and many others, as well as disease states such as hypertension, coronary heart disease, and stroke, are determined by complicated and life-long interactions among multiple genetic and environmental factors. | -complex trait | Weaknesses |
| Brutsaert^44^ | 2006 | What makes a champion? Explaining variation in human athletic performance | This may be because complex traits are fundamentally polygenic (many genes with small effects), or because researchers have failed to take into consideration the full range of environmental effects, or both.  Given the number of SNPs that will have to be genotyped (hundreds of thousands), and the sample sizes that will be required to detect genes of modest effect, the costs of genome-wide association studies will still be enormous.  Additionally, most of the positive results by linkage analysis have not been replicated in association studies. Candidate gene studies have some serious limitations vis-a-vis the goal of elucidating genetic causal pathways. | -complex trait  -costs  -quality | Weaknesses |
| Sharp^45^ | 2008 | The human genome and sport, including epigenetics and athleticogenomics: a brief look at a rapidly changing field | - | - | - |
| McNamee^46^ | 2009 | Genetic testing and sports medicine ethics | - | - | - |
| Ostrander^47^ | 2009 | Genetics of athletic performance | However, with such small numbers it is difficult to draw definitive conclusions, especially as other investigators report conflicting results.  According to their definition, elite athletes are those who have won gold medals at major world competitions including, most notably, the Olympics or world championships, ending with average athletes featured in regional competitions with no less than four years of participation experience. The categorization system, however, is rapidly becoming outdated as sports physiologists and geneticists consider the biological and genetic constitutions of “ultraextreme” athletes who participate in one or more “ultraendurance” races. | -quality | Weaknesses |
| Wackerhage^48^ | 2009 | Genetic research and testing in sport and exercise science: a review of the issues | Most traits will depend on many, often common genetic variants that contribute individually only a few percent at best to the inter-individual variation in these polygenic traits.  The conclusion from the above observations is that studies aimed at discovering novel genotype–phenotype associations are out of reach for most sport and exercise scientists due to the required numbers of participants and costs. | -complex trait  -costs  -quality | Weaknesses |
| Lippi^49^ | 2010 | Genetics and sports | Identifying the relevant genes to human athletic performance has been difficult, in part because each causal gene only makes a small contribution to overall heritability.  Although financial costs are currently a major obstacle, the future prospects are good. | -complex trait  -costs | Weaknesses |
| Rankinen^50^ | 2010 | Advances in exercise, fitness, and performance genomics | Other issues to ponder include population stratification or some other uncontrolled factors, including nonrandom genotyping errors, which could have created a spurious association.  Most studies in the exercise genomic field defined in the broad sense are underpowered and cannot therefore establish a definitive genotype–phenotype relationship. Minimizing the phenotypic error variance (also known as random variance, noise variance) is vital for a successful genetic study. In assessing the quality of a report, one should also consider whether multiple testing was taken into account in reporting and interpreting results.  It is also useful to recognize that there is a strong tendency to publish studies with positive results. Indeed, in the end, very few negative studies reach publication. | -generalizability  -reporting bias  -quality | Weaknesses |
| Bouchard^51^ | 2011 | Overcoming barriers to progress in exercise genomics | It is clear by now that reliance on candidate genes and personal views of what constitutes a valid candidate is insufficient for a solid foundation upon which a complete genomic anatomy of complex traits can be built.  We have to recognize that failure to use an adequate sample size and to adjust the significance level for multiple testing, more than any other factors, are responsible for the lack of reproducibility of exercise genomics findings. | -complex trait  -quality | Weaknesses |
| Bouchard^52^ | 2011 | Genomics and genetics in the biology of adaptation to exercise | As progress is being made in defining the genetic anatomy of complex human traits, such as those of interest to exercise physiologists, it is becoming obvious that a major challenge is and will continue to be understanding what is driving the association between genotype and phenotype.  One potential issue in studies with unrelated subjects that has received a lot of attention is an increased risk of false-positive findings due to population stratification.  A common weakness of the early human exercise genomic studies was that they were based on small sample sizes and thus were grossly underpowered. After a while, it became evident that panels of SNPs and GWASs were not covering well a major fraction of human genomic polymorphisms, which are commonly referred to as copy number variants or CNVs. | -complex trait  -generalizability  -quality | Weaknesses |
| Eynon^53^ | 2011 | Genes and elite athletes: a roadmap for future research | By definition elite athletic phenotype is a complex one; thus, it is likely that the effect of a single gene variant is small.  Between-study differences in the competition level of athletes, sex and ethnic group further complicate this issue.  The polygenic profile was computed assuming (i) an additive effect, and (ii) the fact that all gene variants were given the same weight in the total score. However, whether the selected genes and the gene variants explain the same proportion of the variance in any complex trait is not known.  Studies investigating gene expression analysis are strongly recommended to better understand the molecular mechanisms underlying the association between a given polymorphism and exercise performance phenotypes. Nonetheless, ideally this would require collecting samples from different tissues (e.g. skeletal muscles, myocardium), which might not be feasible in many human subjects, not to mention elite athletes.  The first methodological limitation in the field lies on the fact that genotype:phenotype studies need very large population samples (i.e. hundreds or thousands) to reach sufficient statistical power to allow making solid conclusions. With regards to the latter issue, there is a clear need to replicate association results between genetic polymorphisms and athletic status in populations of different ethnic backgrounds.  As for population selection, some studies report data on athletes of mixed sport disciplines, i.e. not clearly ‘endurance’ or ‘power’ oriented, thereby involving phenotypic heterogeneity.  An additional potential gap to be kept in mind when interpreting the literature in the field is that studies reporting no genetic association with athletic status (i.e. ‘negative results’) are less ‘attractive’ and thus less likely to be published, than others showing ‘statistical significance’ (‘positive results’). | -complex trait  -generalizability  -genotype scores  -invasive methods  -reporting bias  -quality | Weaknesses |
| Hagberg^54^ | 2011 | Advances in exercise, fitness, and performance genomics in 2010 | But there is no other credible solution, particularly in light of the present-day research funding restrictions.  One of the most frequently observed deficiencies of exercise genomics papers is that they are based on small sample sizes and therefore have very limited statistical power to test the hypothesis (or, more commonly, the hypotheses) as defined in their aims statements. | -costs  -quality | Weaknesses |
| Roth^55^ | 2012 | Advances in exercise, fitness, and performance genomics in 2011 | We emphasized the need for appropriately powered studies to address the interactive effects of genetics and exercise training or acute exercise on any complex, multifactorial physiological or pathological phenotype.  While no one can argue with this basic scientific tenet, we recognize that it is commonly difficult, costly, and time- consuming to generate sample sizes of the magnitude required for sufficient statistical power to address genetic questions in acute exercise studies, but even more so in complex exercise training experiments or exercise intervention trials.  A comprehensive picture of the role of genomic differences to the inheritance of exercise-related traits will require that exercise scientists reach beyond common sequence variants and investigate other genomic structures such as rare variants, copy number variants, deletions and insertions, splice site disruptions, mutations impacting microRNAs or other small RNA molecular sequences or binding sequences, premature stop codons, enhancer recognition sequences, and undoubtedly others. | -complex trait  -costs  -quality | Weaknesses |
| Guth^56^ | 2013 | Genetic influence on athletic performance | A primary challenge when attempting to describe the influence of genetic factors on athletic performance is its multifactorial nature. Considering the number of body systems that must interact (musculoskeletal, cardiovascular, respiratory, nervous, etc.), athletic performance is one of the most complex human traits.  Though many specific genes and sequence variants (polymorphisms) within genes have been associated with performance, many of the findings to date have not been adequately replicated. | -complex trait  -quality | Weaknesses |
| Pérusse^57^ | 2013 | Advances in exercise, fitness, and performance genomics in 2012 | - | - | - |
| Wang^58^ | 2013 | Genomics of elite sporting performance: what little we know and necessary advances | World-class athletic performance is a complex multifactorial phenotype, and it is acknowledged that to become an elite athlete, a synergy of physiological, behavioural and other environmental factors is required.  While genetic testing will very likely become a part of talent ID programmes in the future, current genetic testing is of almost zero predictive capacity despite testimonials to the contrary (www.xrgenomics.com/testimonials).  However, most reported associations are cited in studies with small sample sizes without robust replication, and therefore are most likely type 1 errors. Other factors relating to the sample population (family history of traits, ethnically homogeneous populations and a sample size of at least several thousands) and differences in statistical approaches (eg, the conservative Bonferroni correction for multiple testing vs the non-conservative false discovery rate correction or the application of permutation testing approaches) need to be carefully considered to ensure successful application of the GWAS approach. When these criteria are not fulfilled and candidate genes are selected based primarily on the interest of the research group, this approach generates conflicting results with low statistical power and difficulty of being replicated in other populations, and thus low validity.  In addition, the candidate gene approach has largely been implemented in studies with small participant numbers and often without robust replication, perhaps partly attributable to publication bias.  Despite numerous attempts in recent years to discover genetic variants associated with elite athletic performance and, more specifically, elite/world-class athletic status, there has been limited progress owing to few coordinated research efforts involving major funding initiatives/consortia and the reliance on candidate gene analyses, involving a small number of single nucleotide polymorphisms (SNPs) and structural variants (eg, the commonly studied insertion/deletion polymorphisms). GWAS of elite human athletic performance are ongoing, but there are no published papers to date. | -complex trait  -genotype scores  -reporting bias  -research progress  -quality | Weaknesses |
| Breitbach^59^ | 2014 | Conventional and genetic talent identification in sports: will recent developments trace talent? | It is now known that the genetic encoding of athletic performance and its determinants are polygenic. The genetic basis generally involves a complex architecture of the phenotype due to the different numbers of genetic variants influencing the trait, their allele frequencies, effect sizes and multiple gene–gene and gene–environment interactions.  The specificity and sensitivity of several algorithms for predicting variant effects have been rated as being limited, showing a low correlation between predictors from the various algorithms.  This might be explained by the fact that GWA studies do not capture rare variants and show limited statistical power for small gene–gene and gene–environment interactions.  Even if hundreds of thousands of elite athletes existed, the selection of these ‘elites’ from the pool of all athletes in a discipline still requires the definition and assignment of the phenotype of being ‘elite’ and the knowledge of where to draw the line between ‘elite’ and ‘non-elite’. | -complex trait  -genotype scores  -quality | Weaknesses |
| Wolfarth^60^ | 2014 | Advances in exercise, fitness, and performance genomics in 2013 | One clear lesson we have learned is that such traits are much more polygenic in nature than was anticipated even a decade ago. | -complex trait | Weaknesses |
| Ahmetov^19^ | 2015 | Current Progress in Sports Genomics | This may be because complex traits are fundamentally polygenic (numerous genes with small effects) or because researchers failed to take into consideration the full range of environmental effects, or both.  The issues with study designs, sample size, population stratification, and quality of the genotype/phenotype measurement are also of great importance. | -complex trait  -generalizability -quality | Weaknesses |
| Bouchard^17^ | 2015 | Exercise genomics--a paradigm shift is needed: a commentary | All of the above strongly suggest that the relationship between the genome and a given phenotype is highly complex.  In summary, we propose that exercise genomics abandon the current practice of focusing on candidate genes typically defined by authors’ preference or from biases in the published scientific literature, and the reliance on small, statistically underpowered, observational studies. | -complex trait -quality | Weaknesses |
| Bouchard^61^ | 2015 | Personalized preventive medicine: genetics and the response to regular exercise in preventive interventions | - | - | - |
| Loos^62^ | 2015 | Advances in exercise, fitness, and performance genomics in 2014 | These studies often examine outcomes that are very specific and/or complex, such as power, strength, endurance, response to training and elite athlete status, which are assessed in few studies and for which not always agreed upon standardized procedures exist.  This approach is probably the most desirable, but it has the obvious limitation of cost, one that is not likely to be well received by the major funding agencies in the current budgetary climate.  Because of the specificity of the design and outcomes, these studies are much harder to replicate and summary statistics are not easily combined in traditional meta-analyses. | -complex trait  -costs  -quality | Weaknesses |
| Webborn^63^ | 2015 | Direct-to-consumer genetic testing for predicting sports performance and talent identification: Consensus statement | Furthermore they suggest that the evidence of the association between a genetic marker and a trait should be validated at genome-wide significance level (p<5×10−8) in more than one large case–control study and in a cohort of the ethnic/geo- graphic background relevant to the client. This is particularly relevant to talent identification or performance testing where the studies to date are limited in ethnicity and geographic background.  Hence, while there is a little replicated scientific evidence regarding these ACTN3 and ACE polymorphisms on a commercial basis, and one can understand individuals interested in exercise and sport wishing to learn about their own genetic composition within these two well-studied genes, the consensus is that the predictive value of such tests in the context of training responses or talent identification in sport is virtually zero. With regard to predicting future sporting performance, the scientific foundation is extremely limited and largely non-existent. | -generalizability  -genotype scores  -quality | Weaknesses |
| Gibson^64^ | 2016 | Core Concepts in Human Genetics: Understanding the Complex Phenotype of Sport Performance and Susceptibility to Sport Injury | Most complex traits encountered in sport science are assumed to be quantitative traits.  GWASs are much more expensive than CGASs (candidate gene association studies), in part because of the cost of phenotyping and also because of the specialized statistical expertise that is required to assure quality data and to adjust for or eliminate confounding variables.  However, a major challenge faced by these methods is the fact that they require tissue biopsies, ideally more than one in order to compare the transcriptome between different physiologically relevant states (e.g. skeletal muscle prior to training, before exercise and after exercise).  Many genotype-phenotype correlations are published once and never replicated, because replication and real proof often require highly detailed statistical methods applied to a replication cohort with tens of thousands to hundreds of thousands of participants. Other issues such as potential genotyping errors, blinded testing and reporting bias are also worth considering. | -complex trait  -costs  -invasive methods  -reporting bias  -quality | Weaknesses |
| Mattsson^65^ | 2016 | Sports genetics moving forward: lessons learned from medical research | It’s important to emphasize that genetic association, regard- less of how robust, does not infer causality. Causality inference requires specific experimental and analytical methods.  Since sports performance is very complex and a result of a combination of many different traits and aspects, it is necessary to base analyses and conclusions of genetic profiles on more detailed phenotyping.  Sex- and ethnicity-specific analyses will be required to refine sports genetics. To date, the fact that studies of the physiology of sports performance have almost exclusively been conducted on men underlines the importance of studies of both men and women.  The ultimate utility of a genetic test that predicts sports performance will depend on whether enough reliable and robust genetic associations can be found to explain a sufficiently large portion of the overall variability, which at the moment is very far from the case.  While it is possible to sample blood (and to a more limited extent muscle biopsy) multiomic profiles at multiple time points, even from elite athletes, other pertinent human tissues of import (e.g., heart, lung, brain) in the processes of training and athletic fitness are not practically available for research from athletes.  Sports genetics to date has been hampered by small sample sizes and biased methodology, which can lead to erroneous associations and overestimation of effect sizes. Analytically modelling the joint effects of multiple genes or allele features without exhaustive multiple testing is an active area of research. | -causality  -complex trait  -generalizability  -genotype scores  -invasive methods  -quality | Weaknesses |
| Pitsidalis^66^ | 2016 | Athlome Project Consortium: a concerted effort to discover genomic and other "omic" markers of athletic performance | Past reliance on candidate gene studies predominantly focusing on genotyping a limited number of single nucleotide polymorphisms or the insertion/deletion variants in small, often heterogeneous cohorts (i.e., made up of athletes of quite different sport specialties) have not generated the kind of results that could offer solid opportunities to bridge the gap between basic research in exercise sciences and deliverables in biomedicine. | -quality | Weaknesses |
| Sarzynski^67^ | 2016 | Advances in Exercise, Fitness, and Performance Genomics in 2015 | However, lack of substantial funding may be the biggest barrier to such an enterprise, as randomized controlled exercise trials with standardized study designs and measures along with high-throughput technologies and robust computational and bioinformatics tools are costly and beyond the means of any one research site/team. | -costs | Weaknesses |
| Wang^68^ | 2016 | The Future of Genomic Research in Athletic Performance and Adaptation to Training | Athletic performance is a complex trait that requires different genetically driven components of human biological systems to coordinate effectively with the environment so as to excel. It is undoubtedly the case that multiple genes are associated with the performance trait and/or its components, though current progress in understanding the genetic architecture underlying these traits is very limited.  To date, most of the genetic findings in athletic performance have been generated using the candidate gene association approach (in small, often heterogeneous cohorts), and the majority of associations have been inconclusive due primarily to (i) the variants genotyped are not causal and provide incomplete link-age with other functional variants, (ii) studies are underpowered, (iii) population stratification, and (iv) phenotypic and locus heterogeneity. False-positive discoveries can also occur in studies examining multiple genes or splitting the cohorts into subgroups for separate analysis without controlling for multiple testing. | -complex trait  -generalizability  -quality | -Weaknesses |
| Yan^69^ | 2016 | Nature versus Nurture in Determining Athletic Ability | Several genetic variants have been found to be associated with elite performance in the last few years, and the current paradigm is that elite performance is a polygenic trait (i.e. influenced by many gene variants), with minor contributions of each variant to the unique athletic phenotype.  A GWAS of elite human athletic performance is ongoing, but there are no published papers to date. | -complex trait  -research progress | Weaknesses |
| Moran^70^ | 2017 | Tour de France Champions born or made: where do we take the genetics of performance? | Until recently, studies into the genetics of quantitative traits concentrated on common genetic variants; that is variants with a minor allele frequency in the population of >1%.  Furthermore, predisposing variants are often identified in small cohorts without replication of the findings in secondary cohorts. Thus there is a risk that many of the variants identified so far may be false positive artefacts of small cohorts with only the most robustly replicated variants, such as ACTN3 R577X, being true positives. In part, this problem is due to the difficulty in recruiting large enough numbers of elite athletes who are a very small minority of any population. | -complex trait  -quality | Weaknesses |
| Vlahovich^26^ | 2017 | Genetic testing for exercise prescription and injury prevention: AIS-Athlome consortium-FIMS joint statement | Another limitation common to the field of exercise/injury genomics is the cohort homogeneity. The majority of studies in sport and exercise genetics have been conducted using Caucasian/European subjects. An additional consideration in cohort homogeneity is the gene-by-sex interactions.  One of the major limitations in the field of exercise/injury genomics is the relatively low sample size of subjects and/or the general population participating in exercise training studies.  To date, the analysis of single variant candidate genes (often poorly justified) using low-throughput techniques has yielded conflicting findings and inconsistent results. | -generalizability  -quality | Weaknesses |
| Vellers^71^ | 2018 | Inter-individual variation in adaptations to endurance and resistance exercise training: genetic approaches towards understanding a complex phenotype | Furthermore, it is becoming increasingly understood that phenotypes which define exercise training-induced adaptations are complex with numerous structural, functional, physiological, and molecular changes that occur in multiple organs (e.g., heart, skeletal muscle) and systems (cardiovascular, muscular) in response to exercise training.  One key limitation is the lack of a defined phenotype(s) for endurance and resistance training-induced responses, due largely to use of inconsistent research methods among investigators (i.e., modes, frequency, intensity, and duration of study protocols). | -complex trait  -quality | Weaknesses |
| Landen^72^ | 2019 | Genetic and epigenetic sex-specific adaptations to endurance exercise | However, it is important to note that athletic ability is a complex trait that is influenced by many aspects and genetic variants, thus making it challenging to identify variants with large effect sizes.  Most studies investigating the molecular modulators of response to endurance exercise have either solely studied males or combined males and females and adjusted for sex as a confounder in statistical modelling.  A thorough review and a recent commentary on sports genetics highlight the need for larger sample sizes, and both ethnicity-specific and sex-specific analyses to confirm effect sizes of common variants. | -complex trait  -generalizability  -quality | Weaknesses |
| Pickering^73^ | 2019 | The Development of a Personalised Training Framework: Implementation of Emerging Technologies for Performance | As a result of the Larruskain and colleagues study, it is clear that the use of individual pieces of data is likely insufficient in the prediction of complex phenotypes and outcomes, such as injury.  As a result, the accurate determination of epigenetic changes requires the sampling of the specific tissue, such as skeletal muscle, which can be both invasive and traumatic, and hence not palatable to high-level athletes. | -complex trait  -invasive methods | Weaknesses |
| Pickering^74^ | 2019 | Can Genetic Testing Identify Talent for Sport? | As a summary of the above discussion, it seems clear that the provision of elite athlete status is a highly complex, polygenic trait, and that we know very few of the genetic variations that contribute to such a trait.  A further potential roadblock is that there may be different associations between SNPs and elite performance across ethnicities, such that a SNP may be performance enhancing in Caucasians, but not in East Asians, for example, thereby requiring the development of ethnicity-specific SNP panels for the purpose of talent identification.  Earlier work demonstrates that there is considerable similarity in polygenic scores within humans - athletes and non-athletes alike - when a low number of markers (in this case, 22 and 23) are used, such that, again, this approach would likely have limited real world specificity and sensitivity.  They acknowledged their low sample size as a major limiting factor. This limitation is difficult to overcome and represents a significant roadblock in the search for genetic variants associated with elite performance. | -complex trait  -generalizability  -genotype scores  -quality | Weaknesses |
| Gomes^75^ | 2020 | Omics and the molecular exercise physiology | For instance, although some genotypes may correlate with specific phenotypes, traits are not only complex but multifactorial, resulting from the interactions between the genome and the environment.  A significant challenge commonly faced is the lack of reproducibility between studies for various reasons, such as small sample sizes, different methodologies, and various exercise protocols. | -complex trait  -quality | Weaknesses |
| Gray^76^ | 2020 | Utility of genetic testing in athletes for cardiac diseases | - | - | - |
| Naureen^77^ | 2020 | Genetic test for the personalization of sport training | It is worth mentioning that although several studies have reported positive involvement of ACE I/D polymorphism in enhancing endurance and power performance some other studies have failed to report such association which could be due to inclusion of mixed sporting disciplines that results in phenotypic heterogeneity, sample size issues, and other confounding factors such as ethnicity and geography.  The application of TGS however is limited by the fact that it gives same weight to all polymorphisms used. Consequently genetic tests based on one or few genetic markers lack scientific backing for prescription of personalized exercise and sports training.  Even though these tests might provide an insight into individual responses to training and exercise based on the genetic profiles these lack scientific backing unless improved methodologies with much larger sample sizes are used. | -generalizability  -genotype scores  -quality | Weaknesses |
| Tanisawa^9^ | 2020 | Sport and exercise genomics: the FIMS 2019 consensus statement update | Another major problem is the deficiency in how the phenotype is being assessed. The factors shaping athletic performance are diverse. For example, endurance performance that is one of the more simple traits in sports, is shaped by VO2max, VO2 at the lactate threshold, economy of movement and other parameters. However, each physiological marker of performance is also a complex trait, regulated by a network of genes and pathways.  In most case-control studies of elite athletes, the physiological, anthropometric and biomechanical characteristics of the athletes are not well phenotyped. Although a large number of studies have been conducted to identify sport and exercise-related genes, the findings are mostly inconclusive because of a lack of replication. One of the major problems is small sample size. | -complex trait  -quality | Weaknesses |
| Griswold^78^ | 2021 | Using Genomic Techniques in Sports and Exercise Science: Current Status and Future Opportunities | Moreover, unlike single gene traits referenced above, most complex traits are impacted by many common and rare genetic variants affecting the function and/or expression of many genes to varying degrees.  A final underrecognized aspect of sports genomics studies is the prominent role of ancestral diversity in the manifestation of genetic variants in terms of traits and disease.  Results have not been widely replicated and effect sizes have been modest. A limitation of most previous genetic studies in sports medicine to date is statistical power to detect associations given the relatively small sample sizes used. | -complex trait  -generalizability  -quality | Weaknesses |
| Sellami^16^ | 2021 | Molecular Big Data in Sports Sciences: State-of-Art and Future Prospects of OMICS-Based Sports Sciences | - | - | - |
| Wang^79^ | 2021 | Physical Exercise: An Overview of Benefits From Psychological Level to Genetics and Beyond | - | - | - |
| Kim^80^ | 2022 | The genetics of human performance | There is some evidence that genetic ancestry might affect performance — for example, marathon runners of East African ancestry dominate the competition. Furthermore, most studies have focused on men, despite there being strong evidence of differential endurance performance by biological sex.    However, genome-wide association studies (GWAS) of maximal oxygen uptake (VO2max) and elite endurance athletes have yet to yield substantial insight into the genes and mechanisms involved in optimizing performance because of sample size limitations and inconsistent protocols for studying EPA (endurance physical acticity). | -generalizability  -quality | Weaknesses |
| Varillas-Delgado^81^ | 2022 | Genetics and sports performance: the present and future in the identification of talent for sports based on DNA testing | This is certain at least across the normal range of human trait distributions because sports performance phenotypes are complex traits and fundamentally polygenic (numerous genes with small effects) or because researchers failed to take into consideration the full range of environmental effects, or both.  Hence, the possession of a high TGS (total genotype score) for power performance does not guarantee success in power-based sports.  Some of this debate is based on the limitations and methodological issues of the research that presents the evidence on this topic. In this regard, studies investigating the effect of genetics on endurance and power sports performance may be confounded by the low sample sizes, incorrect categorization of elite athletes, the lack of measurement of valid exercise performance traits, and the obtaining of outcomes based only upon cross-sectional observations of genotype frequencies between athlete and non-athlete populations. Candidate gene association studies have often produced inconclusive results. | -complex trait  -genotype scores  -quality | Weaknesses |
| Vicelli^82^ | 2022 | The non-modifiable factors age, gender, and genetics influence resistance exercise | - | - | - |

# Supplementary 7. Content analysis – Opportunities.

| First author | Year | Title | Initial Coding (citations from the articles, chronological order in text may be changed) | Focused  Coding | Theoretical  Coding |
| --- | --- | --- | --- | --- | --- |
| Bray^43^ | 2000 | Genomics, genes, and environmental interaction: the role of exercise | The first-pass sequencing of the human genome is targeted for completion in the spring of 2000, opening up a wealth of new opportunities for gene discovery. | -omics/ technology | Opportunities |
| Brutsaert^44^ | 2006 | What makes a champion? Explaining variation in human athletic performance | High throughput genotyping methods may change the cost equation considerably, and some recent effort has focused on new and more efficient association-based linkage approaches. | -omics/ technology | Opportunities |
| Sharp^45^ | 2008 | The human genome and sport, including epigenetics and athleticogenomics: a brief look at a rapidly changing field | Following from this, a specific training schedule could be designed, which would form the ultimate in personalized coaching – a type of laboratory ‘‘fitness-testing’’ of the genome, paralleling the physiological fitness testing of the body.  Now, the possibilities for gene therapy are excitingly promising, but the possibilities for gene doping are worryingly impending. | -precision exercise/ profiling  -screening/ therapy | Opportunities |
| McNamee^46^ | 2009 | Genetic testing and sports medicine ethics | We note the powerful case that can be made for genetic testing regarding the identification of predisposition to hypertrophic cardiomyopathy. | -screening/ therapy | Opportunities |
| Ostrander^47^ | 2009 | Genetics of athletic performance | - | - | - |
| Wackerhage^48^ | 2009 | Genetic research and testing in sport and exercise science: a review of the issues | Possible applications of genetic research are genetic performance tests or genetic tests to screen, for example, for increased risk of sudden death during sport. | -screening/ therapy | Opportunities |
| Lippi^49^ | 2010 | Genetics and sports | This is a valuable example on how consideration of the genetic variation in disposition of androgens will improve the sensitivity and specificity of the testosterone doping tests.  Genetic testing will indeed help identify individuals with advantageous physiology, morphology and maybe psychology, those with a greater capacity to respond/adapt to training and those with a lower chance of suffering from injuries.  This statement clearly highlights that genetic testing (i) might be useful for the development of genetic performance tests, (ii) may also be applied for pre-participation risk screening and may prevent sudden deaths during sport, (iii) might in future also be used to identify those who are most likely to benefit medically from exercise programmes and (iv) may become more important in anti-doping activities where it could be used for identification purposes (genetic fingerprinting) and more direct antidoping testing. | -anti-doping  -precision exercise/ profiling  -screening/ therapy | Opportunities |
| Rankinen^50^ | 2010 | Advances in exercise, fitness, and performance genomics | - | - | - |
| Bouchard^51^ | 2011 | Overcoming barriers to progress in exercise genomics | Exercise genomics would greatly benefit from the availability of large cohorts of individuals who have been phenotyped for appropriate exercise-related traits. Pooling the data from several cohorts would allow for even greater statistical power to detect loci with small effect sizes.  The next-generation sequencing techniques provide valuable tools to explore common variants, rare variants, CNVs (copy number variants), and other structural variants in relation to complex multifactorial traits of interest to human biologists.  The next generation of exercise guidelines in preventive and therapeutic medicine will have to be grounded in the principles of personalized medicine. | -multicentre studies  -omics/ technology  -precision exercise/ profiling  -screening/ therapy | Opportunities |
| Bouchard^52^ | 2011 | Genomics and genetics in the biology of adaptation to exercise | Systems biology technologies such as transcriptomics, proteomics, and metabolomics are being utilized to deliver a relatively unbiased global profile of how human genes behave under a variety of physiological conditions including exercise training. | -omics/ technology | Opportunities |
| Eynon^53^ | 2011 | Genes and elite athletes: a roadmap for future research | Large collaborations and data sharing between research centres worldwide are strongly recommended.  Elucidating the role of miRNAs in the phenotypic change and individual variability in response to exercise training represents a promising area for further investigation in the field of genetics and athletic performance.  As for predictive algorithms such as the genotype score, several new candidate polymorphisms will likely appear in the foreseeable future, allowing for more accurate predictions. | -multicentre studies  -omics/ technology  -precision exercise/ profiling | Opportunities |
| Hagberg^54^ | 2011 | Advances in exercise, fitness, and performance genomics in 2010 | It would clearly be more productive for the small community of exercise genomics laboratories to work together in order to be able to undertake collaborative studies that would be statistically adequately powered. | -multicentre studies | Opportunities |
| Roth^55^ | 2012 | Advances in exercise, fitness, and performance genomics in 2011 | - | - | - |
| Guth^56^ | 2013 | Genetic influence on athletic performance | The potential for genetic testing to predict injury susceptibility, such as APOE genotype with response to concussion, may provide a unique and important avenue to improve safety for athletes. | -precision exercise/ profiling | Opportunities |
| Pérusse^57^ | 2013 | Advances in exercise, fitness, and performance genomics in 2012 | We again emphasize that it would be advantageous if more studies with appropriate designs and statistical power were undertaken using the latest genomics technologies in sequencing and genotyping, combined with and incorporating evidence from epigenomics, transcriptomics, proteomics, and metabolomics.  It would also make it possible for exercise medicine to become a full partner in the emerging personalized medicine enterprise.  Such a capability would have an enormous impact on our ability to individualize exercise medicine for preventive or therapeutic purposes. | -omics/ technology  -precision exercise/ profiling  -screening/ therapy | Opportunities |
| Wang^58^ | 2013 | Genomics of elite sporting performance: what little we know and necessary advances | This development will require a move away from the traditional way of researching in exercise science/medicine (ie, predominantly single-laboratory studies) to large, well-funded collaborations/consortia with leading industry partners and therefore substantial statistical/ technological power and know-how. | -multicentre studies  -omics/ technology | Opportunities |
| Breitbach^59^ | 2014 | Conventional and genetic talent identification in sports: will recent developments trace talent? | GT (genetic testing) has been supposed to serve in the risk stratification for the participation in high-performance sports instead of GT. DNA analysis might prevent athletes from dying due to exercise without being aware of a serious pathological condition. | -screening/ therapy | Opportunities |
| Wolfarth^60^ | 2014 | Advances in exercise, fitness, and performance genomics in 2013 | We have seen this year (a trend that began a few years ago) several articles genotyping multiple genetic markers, or using GRS (genetic risk scores), or relying on microarray analyses to identify genomic targets, or performing pathway and network analyses on the basis of evidence for genomics or transcriptomics.  Given the potential of these methods to advance our knowledge in exercise biology, it is likely that pathway analyses and other systems biology approaches will become increasingly popular among exercise scientists, especially when genomics and other omics data (transcriptomics, epigenomics, metabolomics, etc.) are incorporated in the models. | -precision exercise/ profiling  -omics/ technology | Opportunities |
| Ahmetov^19^ | 2015 | Current Progress in Sports Genomics | Future research including multicenter GWAS, whole-genome sequencing, epigenetic, transcriptomic, proteomic, and metabolomic profiling and performing meta-analyses in large cohorts of athletes is needed before these findings can be extended to practice in sport.  Accordingly, one of the applications of sports genetics could be the development of predictive genetic performance tests. | -multicentre studies  -precision exercise/ profiling  -omics/ technology | Opportunities |
| Bouchard^17^ | 2015 | Exercise genomics--a paradigm shift is needed: a commentary | The future of exercise genomics lies in large-scale collaborative and multi-centre research programmes.  We recommend that exercise genomic science shifts to unbiased exploration of the genome using all the power of genomics (both GWAS and whole genome sequencing), epigenomics and transcriptomics in combination with large observational (preferably prospective) and experimental study designs, including Mendelian randomisation.  At the other end of the activity spectrum, there are lifelong endurance athletes who are more prone than others to develop cardiac arrhythmias and other cardiac ailments. Being able to identify who is at risk of developing any of these conditions could lead eventually to individualised preventive measures or alternative therapies. | -multicentre studies  -omics/ technology  -screening/ therapy | Opportunities |
| Bouchard^61^ | 2015 | Personalized preventive medicine: genetics and the response to regular exercise in preventive interventions | Though still in its infancy, personalized genomics and related personal omics profiling, encompassing epigenomics, pharmacogenomics, transcriptomics, proteomics, metabolomics, and antibody profiling, will allow for the deep biological profiling of humans in the face of PA (physical activity). | -omics/ technology  -precision exercise/ profiling | Opportunities |
| Loos^62^ | 2015 | Advances in exercise, fitness, and performance genomics in 2014 | Such a comprehensive approach makes it possible in the end to undertake not only a meta-analysis of the findings from each contributing center, but also a mega-analysis as all individual data points have been gathered using exactly the same protocol.  The most convincing observations were reported for GRSs (genomic risk scores), which combine multiple SNPs to assess individuals' genetic susceptibility. As GRSs are continuous exposures that contain more variation than a single SNP, they provide greater statistical power. | -multicentre studies  -precision exercise/ profiling | Opportunities |
| Webborn^63^ | 2015 | Direct-to-consumer genetic testing for predicting sports performance and talent identification: Consensus statement | - | - | - |
| Gibson^64^ | 2016 | Core Concepts in Human Genetics: Understanding the Complex Phenotype of Sport Performance and Susceptibility to Sport Injury | Assessment of the transcriptome offers another approach, whereby the total amount of RNA for a large number of genes is quantified at once. | -omics/ technology | Opportunities |
| Mattsson^65^ | 2016 | Sports genetics moving forward: lessons learned from medical research | Collaboration and collation of larger cohorts, together with improved study design, are required to overcome these limitations.  From a genetics perspective, multiomic profiling of individuals or families with unique athletic attributes may facilitate the discovery or biological validation of genes and/or variants implicated in the genetics of athletic ability.  Once genetic associations and their OR (odds ratio) have been identified in a robust and valid manner, a genetic score can be developed by building a predictive model that includes each genotype, weighted by its effect size. | -multicentre studies  -omics/ technology  -precision exercise/ profiling | Opportunities |
| Pitsidalis^66^ | 2016 | Athlome Project Consortium: a concerted effort to discover genomic and other "omic" markers of athletic performance | Another challenge is to be able to efficiently integrate the multiple “omics” datasets generated from the different approaches.  Finally, identifying genetic markers of exercise capacity, adaptation to exercise programs, and the predisposition to injury is certain to provide useful information to prescribe personalized exercise interventions in the context of 21st-century medicine, which should not be based only on identifying new drug targets but also on implementing lifestyle interventions for disease prevention at the individual level. | -omics/ technology  -precision exercise/ profiling | Opportunities |
| Sarzynski^67^ | 2016 | Advances in Exercise, Fitness, and Performance Genomics in 2015 | The strategy of combining omics with physiological and/or behavioral data seems like a very reasonable approach to increase our understanding of the genes, pathways, and networks contributing to human responsiveness to changes in physical activity behavior and exercise training.  The translation of the findings is obviously a difficult step, but the framework calls mainly for strong randomized controlled trials using Mendelian randomization, with the goal of eventually applying the results in personalized exercise medicine situations. | -omics/ technology  -precision exercise/ profiling | Opportunities |
| Wang^68^ | 2016 | The Future of Genomic Research in Athletic Performance and Adaptation to Training | Therefore, large, collaborative efforts are necessary for meaningful progress to be made in the area of exercise genomics.  In addition, other ‘omics’ approaches, such as epigenomics, transcriptomics, proteomics and metabolomics, will need to be adopted to help dissect the molecular mechanisms of elite athletic performance, response to training, and injury predisposition. | -multicentre studies  -omics/ technology | Opportunities |
| Yan^69^ | 2016 | Nature versus Nurture in Determining Athletic Ability | - | - | - |
| Moran^70^ | 2017 | Tour de France Champions born or made: where do we take the genetics of performance? | To move performance genetics and in particular cycling performance genetics forward, we need: (1) to collect larger cohorts of elite athletes and in particular cyclists as well as persuade them of the importance of collecting detailed phenotypic information (2) to collaborate to pool existing cohorts together; (3) when possible collect additional samples such as blood, plasma and tissue that will allow future epigenetic analyses; (4) to pursue cutting-edge technologies such as next generation sequencing, transcriptomics and proteomics; and (5) to integrate all these results into systems biology models. | -multicentre studies  -omics/ technology | Opportunities |
| Vlahovich^26^ | 2017 | Genetic testing for exercise prescription and injury prevention: AIS-Athlome consortium-FIMS joint statement | The solution is large-scale multi-centre collaborations to drive research in this area and to ensure appropriate cohort size and homogeneity.  Uncovering the genes behind the individual response to exercise training therefore has exciting implications for “personal medicine” and the development of individualised exercise training and health programs.  There is future potential to use genetic screening in assessing risk of musculoskeletal injuries, providing an avenue to modify training, conditioning programs and physical therapy intervention in order to prevent injuries. | -multicentre studies  -precision exercise/ profiling  -screening/ therapy | Opportunities |
| Vellers^71^ | 2018 | Inter-individual variation in adaptations to endurance and resistance exercise training: genetic approaches towards understanding a complex phenotype | Thus, the complexity of physiological and molecular changes occurring with endurance and resistance training stresses the importance of establishing collaborative efforts among investigators which have expertise in the known phenotypic adaptations with endurance and resistance training. | -multicentre studies | Opportunities |
| Landen^72^ | 2019 | Genetic and epigenetic sex-specific adaptations to endurance exercise | Currently, consortia and large-scale studies are underway to advance exercise genomics.  Future studies related to exercise adaptations should integrate genomics, epigenomics, transcriptomics, proteomics, metabolomics, and microbiomics to reveal the underlying molecular mechanisms of sex-specific exercise adaptations.  Elucidating the genetic and epigenetic sex-specific adaptations to exercise is expected to bring highly innovative fundamental knowledge on how individuals respond to exercise, as well as pave the way for future translational studies that are likely to provide evidence-based recommendations regarding personalised health-related interventions. | -multicentre studies  -omics/ technology  -precision exercise/ profiling | Opportunities |
| Pickering^73^ | 2019 | The Development of a Personalised Training Framework: Implementation of Emerging Technologies for Performance | Alongside an understanding of the microbiome, genome and epigenome, along with the utility of other markers such as cfDNA to act as novel markers of exercise adaptation and readiness, there are a variety of other “omes”, including the transcriptome, proteome and metabalome, which may enhance the personalised medicine approach to elite athlete preparation.  Nevertheless, despite these challenges, the development of a personalised training process appears to hold promise in the optimisation of athlete performance. Genetic variation exerts an influence on every aspect of elite athlete performance, including training adaptation, injury risk, ergogenic aid use, post-exercise recovery, athletic development and, at some point, potentially the identification of elite athletes. | -omics/ technology  -precision exercise/ profiling | Opportunities |
| Pickering^74^ | 2019 | Can Genetic Testing Identify Talent for Sport? | As a result, it appears a fundamental requirement that, if genetic testing is to be utilised for talent identification purposes, a far greater number of polymorphisms associated with elite athlete status needs to be uncovered and then combined into a complex TGS (total genotype score) model.  As a result, whilst this information could be used to bias against those with the perceived “unfavourable” genotypes, it could also be used to personalize the training process, identifying those athletes who need greater attention in these areas.  Additionally, genetic information could be utilised to identify those athletes with an increased risk of injury, allowing for the provision of pre-emptive strategies to reduce that risk. | -precision exercise/ profiling  -screening/ therapy | Opportunities |
| Gomes^75^ | 2020 | Omics and the molecular exercise physiology | Hence, it became clear the need for collective efforts to address the interactions between genes and exercise adaptations.  Advances in multi-omics technologies to collect and analyze different longitudinal data have fueled a new health paradigm, where medicine becomes not only more personalized but also more predictive, preventive, and participatory, also known as P4 medicine. | -multicentre studies  -omics/ technology  -precision exercise/ profiling | Opportunities |
| Gray^76^ | 2020 | Utility of genetic testing in athletes for cardiac diseases | - | - | - |
| Naureen^77^ | 2020 | Genetic test for the personalization of sport training | Consequently, one of the most interesting application of sports genetic is development of tests for predicting performance and devise training regime. Furthermore, the potential for genetic testing to predict injury predisposition, may help in ensuring health and safety of athletes during sports training. | -precision exercise/ profiling | Opportunities |
| Tanisawa^9^ | 2020 | Sport and exercise genomics: the FIMS 2019 consensus statement update | There is considerable commercial opportunity associated with the use of genomics in the sport and exercise science.  Integration of various types of genomic variation by multiomics approach will be needed to fully elucidate the complexity of athletic performance. It is likely that elaborate algorithms will be developed using big data processing methods and controlled by the larger companies that have the R&D resources to invest in the necessary analytical technology such as supercomputers, programmers and specialist bioinformaticians.  Identification of specific sport and exercise-related genes are expected to be used for precision sports medicine to provide tailor-made training as well as to select optimal sports and/or other exercise activities for each individual.  In the future, genetic testing may also have a potential role in cardiac screening. | -commercial use  -omics/ technology  -precision exercise/ profiling  -screening/ therapy | Opportunities |
| Griswold^78^ | 2021 | Using Genomic Techniques in Sports and Exercise Science: Current Status and Future Opportunities | Thus, establishment of large collections of sports-related samples is critical. By participating in large, global consortia of clinically well-characterized individuals, genomic studies can increase sample size, represent diverse genetic ancestries, and use cutting edge high-throughput technologies to realize the goal of applying genetics in the form of personalized medicine to the sports medicine clinic.  Aggregation of signals across many studies and samples into polygenic risk scores allow amplification of the relatively small effects of each individual variant and begin to provide evidence for underlying molecular pathway involvement.  Such an approach could enhance our opportunity for development of personalized prevention strategies and novel therapeutic approaches. | -multicentre studies  -omics/ technology  -precision exercise/ profiling  -screening/ therapy | Opportunities |
| Sellami^16^ | 2021 | Molecular Big Data in Sports Sciences: State-of-Art and Future Prospects of OMICS-Based Sports Sciences | The possibility of the influence of other factors and variables not taken into account should also not be ruled out, warranting further high-quality, multi-center research, involving large cohorts of athletes and underlining the importance of large-scale databases and Big Data.  Summarizing, the different OMICS specialties seem to converge in a unique approach, termed sportomics or athlomics and defined as a “holistic and top-down” “non- hypothesis-driven research on an individual’s metabolite changes during sports and exercise” (as stated in the Athlome Project Consortium and in the Santorini Declaration).  Genetics/genomics studies have led to the design of polygenic/ genotype-based scores for profiling athletes in terms of power, sprint, speed, and/or endurance and predict/forecast the effects of training programs and strategies. | -multicentre studies  -omics/ technology  -precision exercise/ profiling | Opportunities |
| Wang^79^ | 2021 | Physical Exercise: An Overview of Benefits From Psychological Level to Genetics and Beyond | More than a dozen genetic variants have been linked to exercise-related traits and outcomes, and this paved way for the development of genetics-based algorithms for personalized training programs. | -precision exercise/ profiling | Opportunities |
| Kim^80^ | 2022 | The genetics of human performance | We strongly advocate for additional multinational, publicly funded projects with similar design to better tease apart the molecular mechanisms through which exercise exerts its beneficial effects.  Unlike GWAS, which focus on fixed DNA associations with outcomes, multi-omic measures are influenced by both underlying genetics and environ-mental exposure; hence, they offer insight into gene–environment and time-dependent interactions.  This diversity is of particular importance as we move towards the implementation of polygenic scores (and other multi-omic measures of risk) in the clinical setting. | -multicentre studies  -omics/ technology  -precision exercise/ profiling | Opportunities |
| Varillas-Delgado^81^ | 2022 | Genetics and sports performance: the present and future in the identification of talent for sports based on DNA testing | These rapid advances in molecular biology techniques open up many possibilities for the development of methods for gene doping detection.  These results open up a path for future research including multicenter GWAS and whole-genome sequencing in large cohorts of athletes with further validation and replication, contributing to the discovery of large numbers of the causal genetic variants (mutations and DNA polymorphisms) that would partly explain the heritability of athlete status and related phenotypes.  A combination of the fields of genomics, epigenomics and transcriptomics along with improved bioinformatic tools, in addition to precise phenotyping, is required for future research to understand the inter-relations of exercise physiology, sports performance and susceptibility to disease.  For this reason, the weighted-total genotype score (w-TGS) model presented by (Varillas Delgado et al. 2020) is a score of genes involved in energy and iron metabolism, suggesting that the relative weighting of the influence of each SNP may add further value in the development of increasingly accurate TGS. | -anti-doping  -multicentre studies  -omics/ technology  -precision exercise/ profiling | Opportunities |
| Vicelli^82^ | 2022 | The non-modifiable factors age, gender, and genetics influence resistance exercise | - | - | - |

# Supplementary 8. Content analysis – Threats.

| First author | Year | Title | Initial Coding (citations from the articles, chronological order in text may be changed) | Focused  Coding | Theoretical  Coding |
| --- | --- | --- | --- | --- | --- |
| Bray^43^ | 2000 | Genomics, genes, and environmental interaction: the role of exercise | - | - | - |
| Brutsaert^44^ | 2006 | What makes a champion? Explaining variation in human athletic performance | - | - | - |
| Sharp^45^ | 2008 | The human genome and sport, including epigenetics and athleticogenomics: a brief look at a rapidly changing field | Now, the possibilities for gene therapy are excitingly promising, but the possibilities for gene doping are worryingly impending. At present, a major worry of the World Anti-Doping Agency is the suspected difficulty in detection, but unsuspected advances in this area may very well occur. | -doping | Threats |
| McNamee^46^ | 2009 | Genetic testing and sports medicine ethics | There are even commercially available test kits for the most eager of sports parents or youth sports coaches with talent identification (and its economic and social benefits) in mind.  Genetic testing for predictive purposes such as talent identification or performance profiling is potentially in breach of the Council of Europe Bioethics Convention and the Genetic Information Non-Discrimination Act in the US.  If genetic testing is to develop in sports medicine, we highlight the need for acceptable and available systems for genetic counselling before and after testing. Moreover, while respect for patient autonomy is often regarded as the crucible of medical ethics, concerns arise from the very nature of genetic data presented under the heading ‘genetic exceptionalism’.  A further problem arises regarding confidentiality: with whom will the genetic data be shared? | -DTC-GT  -ethics | Threats |
| Ostrander^47^ | 2009 | Genetics of athletic performance | For the foreseeable future, detection of gene doping may be impossible. | -doping | Threats |
| Wackerhage^48^ | 2009 | Genetic research and testing in sport and exercise science: a review of the issues | These differences open the door for ‘‘Frankensteinian’’ scenarios where genetic performance tests are performed on embryo DNA to correct a poor genotype by genetic manipulation, to decide whether to have an abortion or to select embryos for implantation. The second potential difference is the possibility of unexpected disease associations. It could be devastating for the individual and could lead to depression or even suicide in those that struggle to cope with the diagnosis. Marriage, employment, life insurance, reproductive choices, and relatives might all be affected. Furthermore, it will be almost impossible to keep such a result confidential, especially among elite athletes given that the athlete will be prevented from competing. | -ethics | Threats |
| Lippi^49^ | 2010 | Genetics and sports | Together with the rapidly increasing knowledge on genetic therapies as a promising new branch of regular medicine, the issue has arisen whether genetics and, especially, gene therapy might be abused in the field of sports. Several issues predict success for this new form of cheating. Transfection of genes virtually identical to those naturally represented in the human genome should outweigh the problem of positive anti-doping testing, making gene doping almost undetectable by traditional laboratory techniques. | -doping | Threats |
| Rankinen^50^ | 2010 | Advances in exercise, fitness, and performance genomics | - | - | - |
| Bouchard^51^ | 2011 | Overcoming barriers to progress in exercise genomics | - |  | - |
| Bouchard^52^ | 2011 | Genomics and genetics in the biology of adaptation to exercise | - | - | - |
| Eynon^53^ | 2011 | Genes and elite athletes: a roadmap for future research | - | - | - |
| Hagberg^54^ | 2011 | Advances in exercise, fitness, and performance genomics in 2010 | - | - | - |
| Roth^55^ | 2012 | Advances in exercise, fitness, and performance genomics in 2011 | - | - | - |
| Guth^56^ | 2013 | Genetic influence on athletic performance | Despite the lack of performance predictability offered by single genetic variants, several companies are marketing genetic tests claiming to do just that. Beyond the oft insufficient rationale for testing these variants, most coaches, parents, and athletes lack the scientific background required to understand the limitations of these tests or the implications of the results.  But, the ethical challenges related to genetic testing in relation to sport performance, especially in children, are especially difficult, as reviewed recently by Wackerhage et al. and need to be considered carefully. | -DTC-GT  -ethics | Threats |
| Pérusse^57^ | 2013 | Advances in exercise, fitness, and performance genomics in 2012 | - | - | - |
| Wang^58^ | 2013 | Genomics of elite sporting performance: what little we know and necessary advances | - | - | - |
| Breitbach^59^ | 2014 | Conventional and genetic talent identification in sports: will recent developments trace talent? | The company, GenEffect, claims that screening the ACTN3 gene indicates whether a person ‘is made’ for sprint, power and strength sport (RR genotype), endurance sport (XX) or mixed pattern sports (RX), although this clear classification has never been demonstrated.  Therefore, genetic tests can be seen as the ‘latest tool’ for parents to pilot their children’s future to an early entrance into professional athletics (whether the children like sports or not). Otherwise, a child being tested negatively for its favourite sport would be excluded from further promotion, even if it willed to train hard. According to ethical standards, these facts reflect a sensitive violation of a person’s autonomy. Further problems arise when a child is genetically tested without full understanding or consent. In particular, whole genome sequencing might simultaneously provide extensive information about disease risk, leading to the question as to whom the results of a genetic test should be provided. | -DTC-GT  -ethics | Threats |
| Wolfarth^60^ | 2014 | Advances in exercise, fitness, and performance genomics in 2013 | - | - | - |
| Ahmetov^19^ | 2015 | Current Progress in Sports Genomics | - | - | - |
| Bouchard^17^ | 2015 | Exercise genomics--a paradigm shift is needed: a commentary | - | - | - |
| Bouchard^61^ | 2015 | Personalized preventive medicine: genetics and the response to regular exercise in preventive interventions | - | - | - |
| Loos^62^ | 2015 | Advances in exercise, fitness, and performance genomics in 2014 | - | - | - |
| Webborn^63^ | 2015 | Direct-to-consumer genetic testing for predicting sports performance and talent identification: Consensus statement | One key concern was the misuse of research evidence and the misinformation about genetic testing, particularly when marketed directly to the public, coaches or parents. This is known as DTC (direct-to-consumer) testing for the purpose of talent identification and to assess potential for future sports performance. Finally, the use of DTC Genetic Testing is irresponsible when it is provided without genetic counselling.  Genetic information is potentially sensitive and as such requires the highest level of security and confidentiality. Genetic information by its very nature means that it is familial. It reveals facts about persons beyond those who have consented to tests, whose results may have direct health implications for other family members. DTC companies must also better address issues around consent, privacy and ownership of data if a company should cease trading or be taken over by a third party. Furthermore the risks of genetic testing for talent identification may not be immediately obvious because the risks may be psychological, social and financial. The psychosocial consequences might include impaired self-esteem, social stigma and, in terms of sport selection, may include employment limitation. | -DTC-GT  -ethics | Threats |
| Gibson^64^ | 2016 | Core Concepts in Human Genetics: Understanding the Complex Phenotype of Sport Performance and Susceptibility to Sport Injury | - | - | - |
| Mattsson^65^ | 2016 | Sports genetics moving forward: lessons learned from medical research | - | - | - |
| Pitsidalis^66^ | 2016 | Athlome Project Consortium: a concerted effort to discover genomic and other "omic" markers of athletic performance | Chief among the concerns, but only one among several, is the problem of consent. | -ethics | Threats |
| Sarzynski^67^ | 2016 | Advances in Exercise, Fitness, and Performance Genomics in 2015 | The entire area of DTC genetic testing for exercise and sports applications is plagued by a score of scientific, ethical, and legal issues, as recently summarized in a consensus report on DTC genetic testing for predicting sports performance and talent identification. Moreover, the results of the DTC genetic tests may have unintended consequences such as undesirable early sport specialization and reduced opportunities for athletic pursuits of youth, not to mention their potential psychosocial and financial implications. Recently, experts in exercise genomics have weighed in on this important topic and provide clear arguments against the current use of genetic testing for athletic performance, especially in children. | -DTC-GT  -ethics | Threats |
| Wang^68^ | 2016 | The Future of Genomic Research in Athletic Performance and Adaptation to Training | Ethical issues around genetic testing are paramount particularly in young children and need to be considered carefully at all times. | -ethics | Threats |
| Yan^69^ | 2016 | Nature versus Nurture in Determining Athletic Ability | - | - | - |
| Moran^70^ | 2017 | Tour de France Champions born or made: where do we take the genetics of performance? | - | - | - |
| Vlahovich^26^ | 2017 | Genetic testing for exercise prescription and injury prevention: AIS-Athlome consortium-FIMS joint statement | There is no role for gene-editing for the purposes of performance enhancement and all genetic manipulations are banned under the World Anti-Doping Agency Code.  A joint FIMS-Athlome Consensus Statement in 2015 warned against the use of DTC tests in athletes, stating that the current level of genetic knowledge is being misrepresented implicitly for commercial purposes and concluding that there is no place for DTC testing for predicting sports performance and talent identification.  Numerous ethical dilemmas exist in relation to genetic testing, for purposes other than medicine. Genomic testing raises a number of important issues for those prescribing the test, including the complexity of informed consent, sample and data storage, return of results, testing involving children, and privacy and confidentiality. A clear process, involving genetic counselling, should be outlined for dealing with unintentional discoveries that may confer a health risk to the participant. | -doping  -DTC-GT  -ethics | Threats |
| Vellers^71^ | 2018 | Inter-individual variation in adaptations to endurance and resistance exercise training: genetic approaches towards understanding a complex phenotype | - | - | - |
| Landen^72^ | 2019 | Genetic and epigenetic sex-specific adaptations to endurance exercise | - | - | - |
| Pickering^73^ | 2019 | The Development of a Personalised Training Framework: Implementation of Emerging Technologies for Performance | - | - | - |
| Pickering^74^ | 2019 | Can Genetic Testing Identify Talent for Sport? | One of the concerns with the use of such DTC genetic testing is that the information provided by the companies is based on a very limited genetic analysis, while the misuse of the research evidence to endorse the utility of this type of testing for early talent selection is common.  Lastly, some variants might also predispose to disease states; a factor raising complex moral and ethical questions. | -DTC-GT  -ethics | Threats |
| Gomes^75^ | 2020 | Omics and the molecular exercise physiology | - | - | - |
| Gray^76^ | 2020 | Utility of genetic testing in athletes for cardiac diseases | There are often significant ethical and legal factors for any athlete considering genetic testing that must be carefully considered prior to proceeding. | -ethics | Threats |
| Naureen^77^ | 2020 | Genetic test for the personalization of sport training | In addition to that, genetic testing of athletes has a potential to be misused by commercial sports companies with preferences being given to some athletes over other thus violating basic human rights.  Genetic testing in sports can raise several ethical concerns related to basic human rights of safety, privacy and secrecy of information. Besides that, the consequence of genetic tests specifically in children that aspire to become athletes can have several negative impacts such as depression and psychological problems in case the sports related genotype is not identified. Furthermore, most coaches, parents and athletes themselves do not have enough scientific background to understands the limitations and implications of results and this raises the questions that who should actually be allowed to ask for a test? | -DTC-GT  -ethics | Threats |
| Tanisawa^9^ | 2020 | Sport and exercise genomics: the FIMS 2019 consensus statement update | Given the development of gene-editing technology such as CRISPR/Cas9, genetic variants determining athletic performance and elite athlete status may be used for gene- doping or creating talented sports children in the future. Designing athletes with extraordinary athletic performance by using gene-editing technique would be a real threat in terms of keeping sport fair, clean and protecting athlete health.  Commercial pressure in most cases results in the premature exploitation of data that have limited or no scientific bases given no or limited replication and validation. Use of such unproven technology can lead to incorrect decisions such as inappropriate early specialisation for sports, inappropriate training, genetic discrimination and even increased health risks.  These rapid advances in DNA sequencing technology have also introduced many new ethical and confidentiality issues such as reidentification of anonymised genotype data, data ownership, newborn screening and incidental findings. | -doping  -DTC-GT  -ethics | Threats |
| Griswold^78^ | 2021 | Using Genomic Techniques in Sports and Exercise Science: Current Status and Future Opportunities | However, as with implementation of genetic technology in any field, many challenges remain before genetic testing has evidence-based practical application in the field of sports medicine. These include the need for adoption of international standards for genomics research and applications and, importantly, recognition and management of agendas driven by direct-to-consumer genetic testing companies and the potential for exploitation for individual or group profit. | -DTC-GT | Threats |
| Sellami^16^ | 2021 | Molecular Big Data in Sports Sciences: State-of-Art and Future Prospects of OMICS-Based Sports Sciences | - | - | - |
| Wang^79^ | 2021 | Physical Exercise: An Overview of Benefits From Psychological Level to Genetics and Beyond | - | - | - |
| Kim^80^ | 2022 | The genetics of human performance | - | - | - |
| Varillas-Delgado^81^ | 2022 | Genetics and sports performance: the present and future in the identification of talent for sports based on DNA testing | There is the possibility of manipulating human genetic material and regulating gene expression to increase or decrease the production of certain enzymes and other proteins associated with processes that are key for human performance. Indeed, until recently, there has been no efficient gene-editing system until the discovery of the CRISPR technique (which shows severe side effects related to gene therapy.  To date, there are serious and well-placed concerns about the use of genetic information, either alone or in combination with existing measures, for talent identification in sport. There remains a lack of universally accepted guidelines and legislation for DTC testing regarding all forms of genetic testing and not just for talent identification. | -doping  -DTC-GT | Threats |
| Vicelli^82^ | 2022 | The non-modifiable factors age, gender, and genetics influence resistance exercise | - | - | - |

DTC-GT=direct-to-consumer genetic testing.

**References**

1. Miyamoto-Mikami E, Zempo H, Fuku N, et al. Heritability estimates of endurance-related phenotypes: A systematic review and meta-analysis. *Scand J Med Sci Sports.* 2018;28(3):834-45.

2. Zempo H, Miyamoto-Mikami E, Kikuchi N, et al. Heritability estimates of muscle strength-related phenotypes: A systematic review and meta-analysis. *Scand J Med Sci Sports.* 2017;27(12):1537-46.

3. Widmann M, Nieß AM, Munz B. Physical Exercise and Epigenetic Modifications in Skeletal Muscle. *Sports Med.* 2019;49(4):509-23.

4. Minchin S, Lodge J. Understanding biochemistry: structure and function of nucleic acids. *Essays biochem.* 2019;63(4):433-56.

5. Robert F, Pelletier J. Exploring the Impact of Single-Nucleotide Polymorphisms on Translation. *Front Genet.* 2018;9:507.

6. Houweling PJ, Papadimitriou ID, Seto JT, et al. Is evolutionary loss our gain? The role of ACTN3 p.Arg577Ter (R577X) genotype in athletic performance, ageing, and disease. *Hum Mutat.* 2018;39(12):1774-87.

7. Baltazar-Martins G, Gutiérrez-Hellín J, Aguilar-Navarro M, et al. Effect of ACTN3 Genotype on Sports Performance, Exercise-Induced Muscle Damage, and Injury Epidemiology. *Sports (Basel).* 2020;8(7).

8. Del Coso J, Hiam D, Houweling P, et al. More than a 'speed gene': ACTN3 R577X genotype, trainability, muscle damage, and the risk for injuries. *Eur J Appl Physiol.* 2019;119(1):49-60.

9. Tanisawa K, Wang G, Seto J, et al. Sport and exercise genomics: the FIMS 2019 consensus statement update. *Br J Sports Med.* 2020;54(16):969-75.

10. Ramaswami R, Bayer R, Galea S. Precision Medicine from a Public Health Perspective. *Annu Rev Public Health.* 2018;39:153-68.

11. Montalvo A, Tse-Dinh Y-C, Liu Y, et al. Precision Sports Medicine: The Future of Advancing Health and Performance in Youth and Beyond. *Strength Cond J.* 2017;39:48-58.

12. Ross R, Goodpaster BH, Koch LG, et al. Precision exercise medicine: understanding exercise response variability. *Br J Sports Med.* 2019;53(18):1141-53.

13. Montgomery HE, Marshall R, Hemingway H, et al. Human gene for physical performance. *Nature.* 1998;393(6682):221-2.

14. Rowe SJ, Tenesa A. Human complex trait genetics: lifting the lid of the genomics toolbox - from pathways to prediction. *Curr Genomics.* 2012;13(3):213-24.

15. Ehlert T, Simon P, Moser DA. Epigenetics in sports. *Sports Med.* 2013;43(2):93-110.

16. Sellami M, Elrayess MA, Puce L, et al. Molecular Big Data in Sports Sciences: State-of-Art and Future Prospects of OMICS-Based Sports Sciences. *Front Mol Biosci.* 2021;8:815410.

17. Bouchard C. Exercise genomics--a paradigm shift is needed: a commentary. *Br J Sports Med.* 2015;49(23):1492-6.

18. Ginevičienė V, Utkus A, Pranckevičienė E, et al. Perspectives in Sports Genomics. *Biomedicines.* 2022;10(2).

19. Ahmetov, II, Fedotovskaya ON. Current Progress in Sports Genomics. *Adv Clin Chem.* 2015;70:247-314.

20. Ahmetov, II, Egorova ES, Gabdrakhmanova LJ, et al. Genes and Athletic Performance: An Update. *Med Sport Sci.* 2016;61:41-54.

21. Ahmetov, II, Hall ECR, Semenova EA, et al. Advances in sports genomics. *Adv Clin Chem.* 2022;107:215-63.

22. Silva HH, Silva MG, Cerqueira F, et al. Genomic profile in association with sport-type, sex, ethnicity, psychological traits and sport injuries of elite athletes. *J Sports Med Phys Fitness.* 2022;62(3):418-34.

23. Yang R, Jin F, Wang L, et al. Prediction and Identification of Power Performance Using Polygenic Models of Three Single-Nucleotide Polymorphisms in Chinese Elite Athletes. *Front Genet.* 2021;12

24. Pranckeviciene E, Gineviciene V, Jakaitiene A, et al. Total genotype score modelling of polygenic endurance-power profiles in Lithuanian elite athletes. *Genes.* 2021;12(7)

25. Guilherme J, Lancha AH, Jr. Total genotype score and athletic status: An exploratory cross-sectional study of a Brazilian athlete cohort. *Ann Hum Genet.* 2020;84(2):141-50.

26. Vlahovich N, Hughes DC, Griffiths LR, et al. Genetic testing for exercise prescription and injury prevention: AIS-Athlome consortium-FIMS joint statement. *BMC Genomics.* 2017;18(Suppl 8):818.

27. Cantelmo RA, da Silva AP, Mendes-Junior CT, et al. Gene doping: Present and future. *Eur J Sport Sci.* 2020;20(8):1093-101.

28. Helms M, Nixon J. Exploring SWOT analysis – where are we now? : A review of academic research from the last decade. *Journal of Strategy and Management - J Econ Manag Strat.* 2010;3:215-51.

29. de-Madaria E, Mira JJ, Carrillo I, et al. The present and future of gastroenterology and hepatology: an international SWOT analysis (the GASTROSWOT project). *Lancet Gastroenterol Hepatol.* 2022;7(5):485-94.

30. Baumeister RF. *Writing a Literature Review.* In: Prinstein MJ. *The Portable Mentor: Expert Guide to a Successful Career in Psychology*. Springer; 2013. 119-32 p.

31. Gates M, Gates A, Guitard S, et al. Guidance for overviews of reviews continues to accumulate, but important challenges remain: a scoping review. *Syst Rev.* 2020;9(1):254.

32. Gates M, Gates A, Pieper D, et al. Reporting guideline for overviews of reviews of healthcare interventions: development of the PRIOR statement. *BMJ.* 2022;378:e070849.

33. Onishi A, Furukawa TA. *State-of-the-Art Reporting*. In: Biondi-Zoccai G. Umbrella Reviews: Evidence Synthesis with Overviews of Reviews and Meta-Epidemiologic Studies. Springer International Publishing; 2016. 189-202 p.

34. Tsagris M, Fragkos KC. *Umbrella Reviews, Overviews of Reviews, and Meta-epidemiologic Studies: Similarities and Differences.* In: Biondi-Zoccai G. *Umbrella Reviews: Evidence Synthesis with Overviews of Reviews and Meta-Epidemiologic Studies.* Springer International Publishing; 2016. 43-54 p.

35. Rethlefsen ML, Kirtley S, Waffenschmidt S, et al. PRISMA-S: an extension to the PRISMA Statement for Reporting Literature Searches in Systematic Reviews. *Syst Rev.* 2021;10(1):39.

36. Franco JVA, Garrote V, Vietto V, et al. Search strategies (filters) to identify systematic reviews in MEDLINE and Embase. *Cochrane Database of Syst Rev.* 2020(7).

37. Salvador-Oliván JA, Marco-Cuenca G, Arquero-Avilés R. Development of an efficient search filter to retrieve systematic reviews from PubMed. *J Med Libr Assoc.* 2021;109(4):561-74.

38. Braun V, Clarke V. Using thematic analysis in psychology*. Qual Res in Psychol.* 2006;3(2):77-101.

39. Corbin J, Strauss A. *Basics of Qualitative Research. Techniques and Procedures for Developing Grounded Theory.* 3^rd^ ed. Sage Publications; 2008.

40. Charmaz K. *Constructing grounded theory: A practical guide through qualitative analysis.* Sage Publications; 2006.

41. Saldaña J. *The coding manual for qualitative researchers.* 4^th^ ed. Sage Publications; 2021.

42. Patel DR, Greydanus DE. Genes and athletes. *Adolesc Med.* 2002;13(2):249-55, v.

43. Bray MS. Genomics, genes, and environmental interaction: The role of exercise. *Jl Appl. Physiol.* 2000;88(2):788-92.

44. Brutsaert TD, Parra EJ. What makes a champion?. Explaining variation in human athletic performance. *Respir Physiol Neurobiol.* 2006;151(2-3):109-23.

45. Sharp NC. The human genome and sport, including epigenetics and athleticogenomics: a brief look at a rapidly changing field. *J Sports Sci.* 2008;26(11):1127-33.

46. McNamee MJ, Müller A, van Hilvoorde I, et al. Genetic testing and sports medicine ethics. *Sports Med.* 2009;39(5):339-44.

47. Ostrander EA, Huson HJ, Ostrander GK. Genetics of athletic performance. *Annual Review of Genomics and Human Genetics.* 2009;10:407-29.

48. Wackerhage H, Miah A, Harris RC, et al. Genetic research and testing in sport and exercise science: a review of the issues. *J Sports Sci.* 2009;27(11):1109-16.

49. Lippi G, Longo UG, Maffulli N. Genetics and sports. *Br Med Bull.* 2010;93:27-47.

50. Rankinen T, Roth SM, Bray MS, et al. Advances in exercise, fitness, and performance genomics. *Med Sci Sports Exerc.* 2010;42(5):835-46.

51. Bouchard C. Overcoming barriers to progress in exercise genomics. *Exerc Sport Sci Rev.* 2011;39(4):212-17.

52. Bouchard C, Rankinen T, Timmons JA. Genomics and genetics in the biology of adaptation to exercise. *Compr Physiol.* 2011;1(3):1603-48.

53. Eynon N, Ruiz JR, Oliveira J, et al. Genes and elite athletes: a roadmap for future research. *J Physiol.* 2011;589(Pt 13):3063-70.

54. Hagberg JM, Rankinen T, Loos RJ, et al. Advances in exercise, fitness, and performance genomics in 2010. *Med Sci Sports Exerc.* 2011;43(5):743-52.

55. Roth SM, Rankinen T, Hagberg JM, et al. Advances in exercise, fitness, and performance genomics in 2011. *Med Sci Sports Exerc.* 2012;44(5):809-17.

56. Guth LM, Roth SM. Genetic influence on athletic performance. *Curr Opin Pediatr.* 2013;25(6):653-58.

57. Pérusse L, Rankinen T, Hagberg JM, et al. Advances in exercise, fitness, and performance genomics in 2012. *Med Sci Sports Exerc.* 2013;45(5):824-31.

58. Wang G, Padmanabhan S, Wolfarth B, et al. Genomics of elite sporting performance: what little we know and necessary advances. *Adv Genet.* 2013;84:123-49.

59. Breitbach S, Tug S, Simon P. Conventional and genetic talent identification in sports: will recent developments trace talent? *Sports Med.* 2014;44(11):1489-503.

60. Wolfarth B, Rankinen T, Hagberg JM, et al. Advances in exercise, fitness, and performance genomics in 2013. *Med Sci Sports Exerc.* 2014;46(5):851-9.

61. Bouchard C, Antunes-Correa LM, Ashley EA, et al. Personalized preventive medicine: genetics and the response to regular exercise in preventive interventions. *Prog Cardiovasc Dis.* 2015;57(4):337-46.

62. Loos RJ, Hagberg JM, Pérusse L, et al. Advances in exercise, fitness, and performance genomics in 2014. *Med Sci Sports Exerc.* 2015;47(6):1105-12.

63. Webborn N, Williams A, McNamee M, et al. Direct-to-consumer genetic testing for predicting sports performance and talent identification: Consensus statement. *Br J Sports Med.* 2015;49(23):1486-91.

64. Gibson WT. Core Concepts in Human Genetics: Understanding the Complex Phenotype of Sport Performance and Susceptibility to Sport Injury. *Med Sport Sci.* 2016;61:1-14.

65. Mattsson CM, Wheeler MT, Waggott D, et al. Sports genetics moving forward: lessons learned from medical research. *Physiol Genomics.* 2016;48(3):175-82.

66. Pitsiladis YP, Tanaka M, Eynon N, et al. Athlome Project Consortium: a concerted effort to discover genomic and other "omic" markers of athletic performance. *Physiol Genomics.* 2016;48(3):183-90.

67. Sarzynski MA, Loos RJ, Lucia A, et al. Advances in Exercise, Fitness, and Performance Genomics in 2015. *Med Sci Sports Exerc.* 2016;48(10):1906-16.

68. Wang G, Tanaka M, Eynon N, et al. The Future of Genomic Research in Athletic Performance and Adaptation to Training. *Med Sport Sci.* 2016;61:55-67.

69. Yan X, Papadimitriou I, Lidor R, et al. Nature versus Nurture in Determining Athletic Ability. *Med Sport Sci.* 2016;61:15-28.

70. Moran CN, Pitsiladis YP. Tour de France Champions born or made: where do we take the genetics of performance? *J Sports Sci.* 2017;35(14):1411-19.

71. Vellers HL, Kleeberger SR, Lightfoot JT. Inter-individual variation in adaptations to endurance and resistance exercise training: genetic approaches towards understanding a complex phenotype. *Mamm Genome.* 2018;29(1-2):48-62.

72. Landen S, Voisin S, Craig JM, et al. Genetic and epigenetic sex-specific adaptations to endurance exercise. *Epigenetics.* 2019;14(6):523-35.

73. Pickering C, Kiely J. The Development of a Personalised Training Framework: Implementation of Emerging Technologies for Performance. *J Funct Morphol Kinesiol.* 2019;4(2):25.

74. Pickering C, Kiely J, Grgic J, et al. Can genetic testing identify talent for sport? *Genes.* 2019;10(12).

75. Gomes C, Almeida JA, Franco OL, et al. Omics and the molecular exercise physiology. *Adv Clin Chem.* 2020;96:55-84.

76. Gray B, Semsarian C. Utility of genetic testing in athletes. *Clin Cardiol.* 2020;43(8):915-20.

77. Naureen Z, Perrone M, Paolacci S, et al. Genetic test for the personalization of sport training. *Acta Biomedica.* 2020;9:1-15.

78. Griswold AJ, Correa D, Kaplan LD, et al. Using Genomic Techniques in Sports and Exercise Science: Current Status and Future Opportunities. *Curr Sports Med Rep.* 2021;20(11):617-23.

79. Wang Y, Ashokan K. Physical Exercise: An Overview of Benefits From Psychological Level to Genetics and Beyond. *Front Physiol.* 2021;12.

80. Kim DS, Wheeler MT, Ashley EA. The genetics of human performance. *Nat Rev Genet.* 2022;23(1):40-54.

81. Varillas-Delgado D, Del Coso J, Gutierrez-Hellin J, et al. Genetics and sports performance: the present and future in the identification of talent for sports based on DNA testing. *Eur J Appl Physiol.* 2022;16.

82. Viecelli C, Ewald CY. The non-modifiable factors age, gender, and genetics influence resistance exercise. *Front Aging.* 2022;3:1005848.

83. Eynon N, Hanson ED, Lucia A, et al. Genes for elite power and sprint performance: ACTN3 leads the way. *Sports Med.* 2013;43(9):803-17.

84. Maffulli N, Margiotti K, Longo UG, et al. The genetics of sports injuries and athletic performance. *Muscles Ligaments Tendons J.* 2013;3(3):173-89.

85. Tucker R, Santos-Concejero J, Collins M. The genetic basis for elite running performance. *Br J Sports Med.* 2013;47(9):545-9.

86. Vancini RL, Pesquero JB, Fachina RJ, et al. Genetic aspects of athletic performance: the African runners phenomenon. *Open Access J Sports Med.* 2014;5:123-7.

87. Heffernan SM, Kilduff LP, Day SH, et al. Genomics in rugby union: A review and future prospects. *Eur J Sport Sci.* 2015;15(6):460-68.

88. Lundby C, Montero D, Joyner M. Biology of VO(2) max: looking under the physiology lamp. *Acta Physiol (Oxf).* 2017;220(2):218-28.

89. Southward K, Rutherfurd-Markwick K, Badenhorst C, et al. The Role of Genetics in Moderating the Inter-Individual Differences in the Ergogenicity of Caffeine. *Nutrients.* 2018;10(10).

90. Costello DM, Kaye AH, O'Brien TJ, et al. Sport related concussion - Potential for biomarkers to improve acute management. *J Clin Neurosci.* 2018;56:1-6.

91. Brazier J, Antrobus M, Stebbings GK, et al. Tendon and Ligament Injuries in Elite Rugby: The Potential Genetic Influence. *Sports (Basel).* 2019;7(6).

92. Herbert AJ, Williams AG, Hennis PJ, et al. The interactions of physical activity, exercise and genetics and their associations with bone mineral density: implications for injury risk in elite athletes. *Eur J Appl Physiol.* 2019;119(1):29-47.

93. Antrobus MR, Brazier J, Stebbings GK, et al. Genetic Factors That Could Affect Concussion Risk in Elite Rugby. *Sports (Basel).* 2021;9(2).

94. Barreto G, Grecco B, Merola P, et al. Novel insights on caffeine supplementation, CYP1A2 genotype, physiological responses and exercise performance. *Eur J Appl Physiol.* 2021;121(3):749-69.

95. Mareddy C, Sc MM, McDaniel G, et al. Exercise in the Genetic Arrhythmia Syndromes - A Review. *Clin Sports Med.* 2022;41(3):485-510.

96. Meyler S, Bottoms L, Muniz-Pumares D. Biological and methodological factors affecting V̇O2max response variability to endurance training and the influence of exercise intensity prescription. *Exp Physiol.* 2021;106(7):1410-24.

97. Cabrera-Serrano M, Ravenscroft G. Recent advances in our understanding of genetic rhabdomyolysis. *Curr Opin Neurol.* 2022;35(5):651-57.

98. Bassini A, Cameron LC. Sportomics: building a new concept in metabolic studies and exercise science. *Biochem Biophys Res Commun.* 2014;445(4):708-16.

99. Kennedy AD, Wittmann BM, Evans AM, et al. Metabolomics in the clinic: A review of the shared and unique features of untargeted metabolomics for clinical research and clinical testing. *J Mass Spectrom.* 2018;53(11):1143-54.

100. Hill BG, Shiva S, Ballinger S, et al. Bioenergetics and translational metabolism: Implications for genetics, physiology and precision medicine. *Biol. Chem.* 2019.

101. Polli A, Ickmans K, Godderis L, et al. When Environment Meets Genetics: A Clinical Review of the Epigenetics of Pain, Psychological Factors, and Physical Activity. *Arch Phys Med Rehabil.* 2019;100(6):1153-61.

102. Hall ECR, Murgatroyd C, Stebbings GK, et al. The Prospective Study of Epigenetic Regulatory Profiles in Sport and Exercise Monitored Through Chromosome Conformation Signatures. *Genes (Basel).* 2020;11(8).

103. Kelly RS, Kelly MP, Kelly P. Metabolomics, physical activity, exercise and health: A review of the current evidence. *Biochim Biophys Acta Mol Basis Dis.* 2020;1866(12).

104. Malsagova KA, Butkova TV, Kopylov AT, et al. Molecular portrait of an athlete. *Diagnostics (Basel).* 2021;11(6).

105. Nieman DC. Multiomics Approach to Precision Sports Nutrition: Limits, Challenges, and Possibilities. *Front Nutr.* 2021;8:796360.

106. Schneider AJ, Fedoruk MN, Rupert JL. Human genetic variation: new challenges and opportunities for doping control. *J Sports Sci.* 2012;30(11):1117-29.

107. Fischetto G, Bermon S. From gene engineering to gene modulation and manipulation: can we prevent or detect gene doping in sports? *Sports Med.* 2013;43(10):965-77.

108. Unal M, Ozer Unal D. Gene doping in sports. *Sports Med.* 2004;34(6):357-62.

109. Gaffney GR, Parisotto R. Gene Doping: A Review of Performance-Enhancing Genetics. *Pediatr Clin North Am.* 2007;54(4):807-22.

110. Zhang X, Speakman JR. Genetic factors associated with human physical activity: Are your genes too tight to prevent you exercising? *Endocrinology.* 2019;160(4):840-52.

111. Gordon ES, Gordish Dressman HA, Hoffman EP. The genetics of muscle atrophy and growth: the impact and implications of polymorphisms in animals and humans. *Int J Biochem Cell Biol.* 2005;37(10):2064-74.

112. Sarpeshkar V, Bentley DJ. Adrenergic-β2 receptor polymorphism and athletic performance. *J Hum Genet.* 2010;55(8):479-85.

113. Angelopoulos TJ, Lowndes J, Seip RL. Genetics of the adaptation to exercise. *World Rev Nutr Diet.* 2011;102:144-49.

114. Collins M, O''Connell K, Posthumus M. Genetics of Musculoskeletal Exercise-Related Phenotypes. *Med Sport Sci.* 2016;61:92-104.

115. Venezia AC, Roth SM. Recent Research in the Genetics of Exercise Training Adaptation. *Med Sport Sci.* 2016;61:29-40.

116. Pickering C, Kiely J. ACTN3: More than Just a Gene for Speed. *Front Physiol.* 2017;8:1080.

117. van de Vegte YJ, Tegegne BS, Verweij N, et al. Genetics and the heart rate response to exercise. *Cell Mol Life Sci.* 2019.

118. Rankinen T, Bray MS, Hagberg JM, et al. The human gene map for performance and health-related fitness phenotypes: the 2005 update. *Med Sci Sports Exerc.* 2006;38(11):1863-88.

119. Alvarez-Romero J, Voisin S, Eynon N, et al. Mapping Robust Genetic Variants Associated with Exercise Responses. *Int J Sports Med.* 2021;42(1):3-18.

120. Balberova OV, Bykov EV, Medvedev GV, et al. Candidate genes of regulation of skeletal muscle energy metabolism in athletes. *Genes.* 2021;12(11).

121. Konopka MJ, van den Bunder J, Rietjens G, et al. Genetics of long-distance runners and road cyclists-A systematic review with meta-analysis. *Scand J Med Sci Sports.* 2022.

122. Mohan R. A Mini Review on Sports Genetics Researches in India: Where We Stand Now. *IIOAB Journal.* 2022;13(1):1-3.

123. Pickering C, Kiely J. Can Genetic Testing Predict Talent? A Case Study of 5 Elite Athletes. *Int J Sports Physiol Perform.* 2020;16(3):429-34.

124. Williams AG, Folland JP. Similarity of polygenic profiles limits the potential for elite human physical performance. *J Physiol.* 2008;586(1):113-21.

125. Lightfoot JT, Roth SM, Hubal MJ. Systems Exercise Genetics Research Design Standards. *Med Sci Sports Exerc.* 2021;53(5):883-87.

126. Nowell LS, Norris JM, White DE, et al. Thematic Analysis:Striving to Meet the Trustworthiness Criteria. *Int J Qual Methods.* 2017;16(1):1609406917733847.

127. Foundation ES, Academies AE. *The European code of conduct for research integrity.* European Science Foundation; 2011.

128. Yuan Y, Hunt RH. Systematic reviews: the good, the bad, and the ugly. *Am J Gastroenterol.* 2009;104(5):1086-92.

129. Maciejewska-Skrendo A, Cięszczyk P, Chycki J, et al. Genetic Markers Associated with Power Athlete Status. *J Hum Kinet.* 2019;68:17-36.

130. Youn BY, Ko SG, Kim JY. Genetic basis of elite combat sports athletes: a systematic review. *Biol Sport.* 2021;38(4):667-75.

131. Bye A, Klevjer M, Ryeng E, et al. Identification of novel genetic variants associated with cardiorespiratory fitness. *Prog Cardiovasc Dis.* 2020;63(3):341-49.

132. Appel M, Zentgraf K, Krüger K, et al. Effects of Genetic Variation on Endurance Performance, Muscle Strength, and Injury Susceptibility in Sports: A Systematic Review. *Front Physiol.* 2021;12:694411.

133. Lim T, Santiago C, Pareja-Galeano H, et al. Genetic variations associated with non-contact muscle injuries in sport: A systematic review. *Scand J Med Sci Sports.* 2021;31(11):2014

134. Guest NS, Horne J, Vanderhout SM, et al. Sport Nutrigenomics: Personalized Nutrition for Athletic Performance. *Front Nutr.* 2019;6:8.
